# Reductant-Responsive Squalene Dual-Prodrug Nanoparticles Co-Delivering Doxorubicin and Exatecan for Synergistic Breast Tumor Therapy

**DOI:** 10.3390/pharmaceutics18091155

**Published:** 2026-09-15

**Authors:** Li Wang, Ning Yang, Yina Sa, Zhenghua Li, Yanlong Qiao, Yanxin Yang, Lixue Chen, Xiangyu Liu, Lei Li

**Affiliations:** 1 College of Pharmacy, Dalian Medical University, Dalian 116044, China; 2 Department of Day Surgery, The First Affiliated Hospital of Dalian Medical University, Dalian 116021, China

**Keywords:** squalene prodrug, doxorubicin, exatecan, reductant-responsive release, synergistic chemotherapy

## Abstract

**Background**: combination chemotherapy employing agents with complementary mechanisms of action can enhance therapeutic efficacy and overcome resistance, but mismatched pharmacokinetics and systemic toxicity limit benefit. **Methods**: We designed reductant-responsive squalene prodrugs of doxorubicin (DOX) and exatecan (EXA) that co-assemble with DSPE-PEG_2k_ into approximately 76 nm dual-prodrug nanoparticles (doxorubicin prodrug–exatecan prodrug nanoparticles, DP-EP NPs) at a predefined synergistic ratio 1:2. **Results**: The minimalist, drug-as-carrier formulation achieved high effective loadings with minimal leakage under normoxia, yet synchronously liberated both drugs in reductive, hypoxia-mimicking media (24 h release: DOX ~65% and EXA ~58% at 1 mM Na_2_S_2_O_4_ vs. ~8–11% at 0 mM Na_2_S_2_O_4_). In MCF-7 and 4T1 cells, the nanoparticles enhanced intracellular accumulation, intensified the disruption of DNA damage repair, as evidenced by increased colocalization of 53BP1 and enhanced γ-H2AX foci, and induced markers of immunogenic cell death, including increased CRT-associated cellular fluorescence and reduced intracellular HMGB1 staining. Furthermore, they partially reversed drug resistance in MCF-7/ADR cells. In rats, the formulation prolonged circulation and increased exposure (DOX t_½_ ~9×, AUC ~1.8×; EXA t_½_ ~5×, AUC ~14.9×) relative to the free-drug mixture. In 4T1 tumor-bearing mice, the nanoparticles produced the greatest tumor-growth inhibition with improved tolerability, supported by stable body weight and benign histopathology. **Conclusions**: These findings establish a scalable, excipient-lean platform that aligns pharmacokinetics with microenvironment-triggered pharmacodynamics to deliver synchronized Topo II/I inhibition for synergistic chemotherapy.

## 1. Introduction

Combination chemotherapy is a cornerstone of solid-tumor management [1,2,3], yet durable responses remain limited by (i) pharmacokinetic (PK) mismatch between agents, which undermines schedule- and ratio-dependent synergy; (ii) dose-limiting systemic toxicities that preclude treatment intensification; and (iii) tumor-microenvironmental barriers—hypoxia, aberrant vasculature, and elevated interstitial pressure—that impede homogeneous intratumoral drug distribution [4,5,6]. Anthracyclines such as doxorubicin (DOX, topoisomerase II inhibitor) and Camptothecin derivatives such as exatecan (EXA, topoisomerase I inhibitor) target complementary nodes of DNA damage and replication stress [7,8,9]. Preclinical evidence suggests that co-inhibition of Topo I/II can forestall compensatory DNA-repair pathways and overcome resistance [10,11]. However, free-drug co-administration typically yields discordant plasma half-lives, rapid renal/hepatic clearance, and non-overlapping biodistribution, making it difficult to maintain synergistic exposure ratios in tumors while respecting safety margins [12,13].

Stimuli-responsive nanomedicine provides an avenue to coordinate pharmacokinetics with spatiotemporally controlled release [14,15]. Tumor hypoxia is a pervasive, spatially heterogeneous characteristic arising from disorganized vasculature and oxygen consumption; it is linked to therapy resistance and immune suppression but also offers a biochemical trigger for selective activation [16,17,18]. Azo linkers undergo reductive cleavage via enzymatic and non-enzymatic reductants, which are enriched in hypoxic tumor microenvironments. This mechanism allows for localized payload release while minimizing impact on normoxic tissues [19]. Nevertheless, numerous carrier-based platforms continue to be constrained by limited loading capacity, compositional complexity that impedes scale-up, and premature leakage that compromises site-specific activation.

Lipidation with small molecules offers a minimalist strategy that transforms the parent drug into an amphiphilic prodrug, enabling self-assembly and achieving high effective loading [20,21]. Squalene (SQ), a biogenic triterpene intermediate in cholesterol biosynthesis that exhibits pronounced dynamic folding, has emerged as a versatile hydrophobe for constructing solvent-lean nanoassemblies: conjugation to SQ promotes hydrophobic association and nanoprecipitation into kinetically stable particles; co-assembly with a small fraction of PEGylated lipids (e.g., DSPE-PEG2k) improves colloidal stability, circulation time, and functional modification [22,23,24,25].

Previous studies have demonstrated that conjugation with squalene can promote the self-assembly of individual therapeutic agents into nanoparticles, as exemplified by squalenoyl–doxorubicin and enzyme-responsive squalenoyl–chidamide systems [23,26]. Recent hypoxia-responsive nanomedicines have also employed azo-based linkers to activate a single chemotherapeutic agent, frequently in combination with photodynamic therapy or other externally activated modalities [27,28]. In addition, dual-prodrug co-assembly has been explored using polymeric conjugates responsive to tumor-associated enzymes [29]. However, these systems generally do not simultaneously integrate two complementary cytotoxic drugs as self-assembling, hypoxia-responsive squalene prodrugs or evaluate their coordinated pharmacokinetic behavior. Therefore, the advancement of the present system does not rely on squalenoylation or azo chemistry individually, but on their integration with synergy-guided drug selection, dual-prodrug co-assembly at a predefined synergistic ratio, concurrent reductant-responsive drug release, and dual-analyte pharmacokinetic assessment.

Building on these principles, we designed dual squalene–prodrug nanoparticles that co-deliver DOX and EXA using a hypoxia-cleavable azo linker on each cargo. Our working hypothesis was that (1) co-assembly of DOX-PRO and EXA-PRO at a predefined synergistic ratio would synchronize PK and intratumoral exposure, (2) hypoxia-triggered cleavage would enable concurrent release of Topo I/II inhibitors precisely in oxygen-deprived tumor regions, and (3) the minimalist composition (drug-as-carrier, DSPE-PEG_2k_ as a stabilizer) would afford high loading and robust manufacturability.

**Figure 1:**
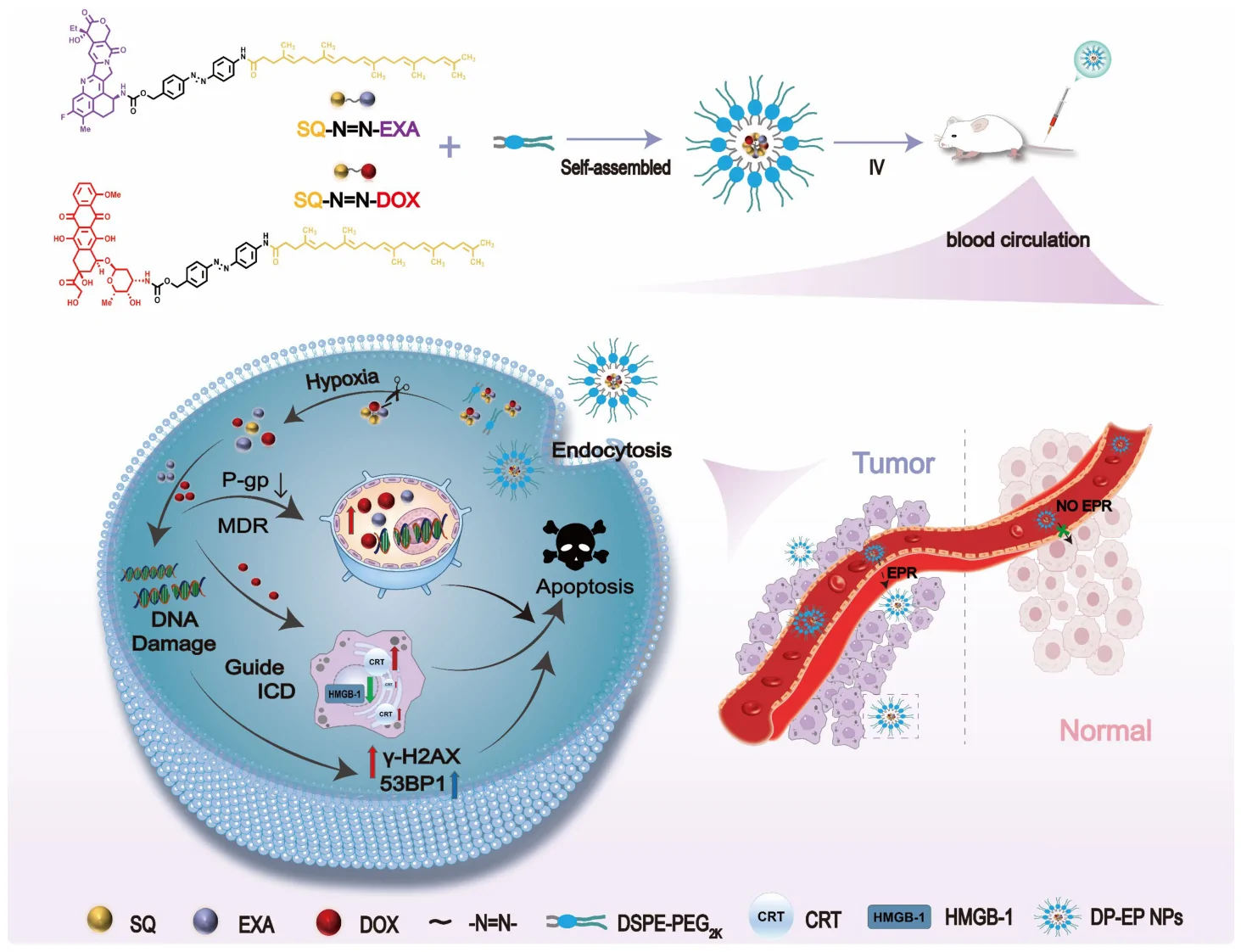
Schematic illustration of the hypoxia-responsive squalenyl DOX and EXA prodrugs, their co-assembly into DP-EP NPs, and their proposed intracellular actions.

This study (i) established a complete formulation-to-mechanism pipeline, including synergy screening, ratio-controlled co-assembly, and hypoxia-triggered release profiling; (ii) demonstrated coordinated pharmacological effects in vitro across scales, including cellular uptake, DNA damage and repair interference, and immunogenic cell death (ICD) markers; (iii) quantified PK advantages in rats with prolonged exposure and reduced clearance for both drugs; and (iv) validated superior antitumor efficacy and tolerability in the 4T1 breast-tumor model. Collectively, our results support a broadly extensible strategy for aligning combination-therapy pharmacology with microenvironment-specific activation while simplifying composition for translational development (Figure 1).

## 2. Materials and Methods

### 2.1. Chemicals and Materials

Doxorubicin hydrochloride was purchased from Energy Chemical (Shanghai, China), and exatecan was obtained from MedChemExpress (Monmouth Junction, NJ, USA). Squalene or squalenyl acid and Na_2_S_2_O_4_ were purchased from Shanghai Aladdin Biochemical Technology, Shanghai, China. tert-Butyldimethylsilyl chloride (TBDMSCl), 1-ethyl-3-(3-dimethylaminopropyl)carbodiimide hydrochloride (EDCI·HCl), and anhydrous solvents were obtained from Anhui Zesheng Technology, Anqing, China. DSPE-PEG2k was purchased from Sigma-Aldrich, Burlington, MA, USA. Fetal bovine serum, DMEM, and RPMI-1640 were supplied by Beijing Solarbio Science & Technology, Beijing, China. CCK-8 reagent was obtained from Suzhou Uelandy Biotechnology, Suzhou, China. Antibodies against γ-H2AX, 53BP1, calreticulin, and HMGB1 were purchased from Wuhan Sanying Biotechnology, Wuhan, China. Unless otherwise specified, all reagents were used as received.

### 2.2. Synthesis of DOX-PRO and EXA-PRO

**Scheme 1:**
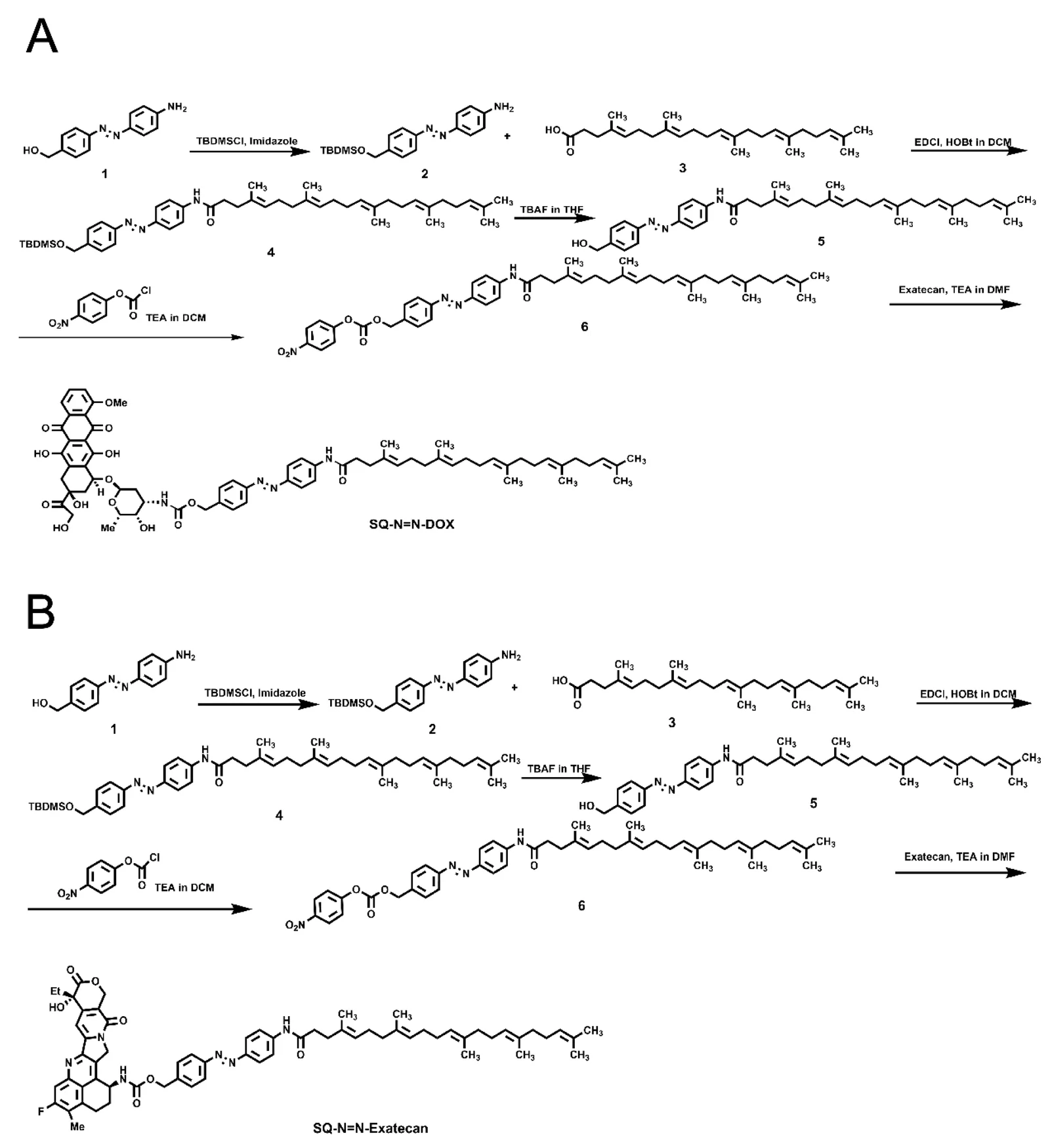
The synthetic route of DOX prodrug (**A**) and EXA prodrug (**B**).

The synthesis sequence was outlined in Scheme 1. To a chilled solution (0 °C) of compound **2** (20.0 mmol) and imidazole (30.0 mmol) in THF was added a THF solution of TBDMSCl (20.0 mmol). The resulting mixture was then stirred at ambient temperature for 6 h. After removing THF under reduced pressure, the residue was taken up in DCM. The resulting compound **3** was carried forward without further purification. Under an argon atmosphere, a mixture of **3** (341 mg, 1.0 mmol), **4** [30] (400 mg, 1.0 mmol), EDCI (230 mg, 1.2 mmol), a catalytic amount of DMAP, and anhydrous DCM (20 mL) was stirred overnight at room temperature. The reaction mixture was then washed with dilute hydrochloric acid (20 mL), and the organic phase was dried over anhydrous Na_2_SO_4_. Concentration under vacuum gave a crude residue, which was purified by column chromatography (PE:AcOEt = 10:1) to afford **5**. A THF solution of TBAF was added dropwise to a solution of **5** in THF at room temperature. After 2 h, analysis confirmed complete deprotection of the TBDMS group. Following evaporation of THF, compound **6** was obtained cleanly without detectable byproducts. In an ice bath, a solution of acyl chloride in anhydrous DCM was added dropwise over 30 min to a mixture of **6** and TEA, maintaining the temperature near 0°C. The resulting salts were removed by washing with water, and the organic layer was concentrated. Purification of the crude material by column chromatography (PE:AcOEt = 5:1) furnished the key intermediate **7**. Finally, a mixture of **7**, TEA, and hydrogen chloride in dioxane in DMF was stirred overnight at room temperature, then diluted with DCM. The reaction mixture was directly loaded onto a silica gel column, and elution with DCM:MeOH = 10:1 afforded the target compound **1a** (DOX-PRO) after removal of the organic solvent. ^1^H NMR (DMSO-*d*_6_, 400 MHz) 13.97 (1H, s), 13.22 (1H, s), 10.20 (1H, s), 7.81–7.76 (8H, m), 7.53 (1H, d, *J* = 5.20 Hz), 7.47 (2H, d, *J* = 7.88 Hz), 5.42 (1H, s), 5.22 (1H, s), 5.17–4.99 (8H, m), 4.9 (1H, bro. s), 4.85 (1H, t, *J* = 5.76 Hz), 4.73 (1H, d, *J* = 5.24 Hz), 4.59 (2H, d, *J* = 5.40 Hz), 4.18 (1H, d, *J* = 6.48 Hz), 3.93 (3H, s), 3.75 (1H, bro. s), 3.48 (1H, bro. s), 3.00–2.89 (2H, m), 2.43 (2H, t, *J* = 7.08 Hz), 2.27 (2H, t, *J* = 6.68 Hz), 2.01–1.89 (18H, m), 1.60 (8H, bro. s), 1.52 (12H, s), 1.34 (3H, d, *J* = 5.92 Hz) (Figure S1). MS [M+H]^+^ = 1179.7, [M+Na]^+^ = 1201.7 (Figure S2).

After preparing **7**, at room temperature, hydrogen chloride EXA, **7**, TEA in DMF, the mixture was stirred overnight, then diluted with water. The precipitation can be collected to get the terminal compound **1b** (EXA-PRO) without further purification. ^1^H NMR (DMSO-*d*_6_, 400 MHz) 10.21 (1H, s), 8.15 (1H, d, *J* = 8.28 Hz), 7.84–7.77 (6H, m), 7.71 (1H, d, *J* = 10.8 Hz), 7.60 (2H, d, *J* = 7.72 Hz), 7.29 (1H, s), 6.51 (1H, s), 5.42 (2H, dd, *J* = 17.10, 3.96 Hz), 5.32–5.10 (6H, m), 5.10–4.96 (4H, m), 3.28–3.15 (1H, m), 3.15–3.00 (1H, m), 2.43 (2H, t, *J* = 7.60 Hz), 2.32 (3H, s), 2.27 (2H, t, *J* = 7.60 Hz), 2.24–2.13 (2H, m), 2.08–1.78 (18H, m), 1.68–1.43 (18H, m), 0.86 (3H, t, *J* = 7.12 Hz) (Figure S3). HR MS [M+H]^+^ = 1071.5776 (Figure S4).

In summary, for DOX-PRO, the ^1^H NMR spectrum and mass spectrum showed the expected signals and molecular ions at *m*/*z* 1179.7 [M+H]^+^ and 1201.7 [M+Na]^+^, respectively. For EXA-PRO, structural confirmation was obtained by ^1^H NMR and high-resolution mass spectrometry, with the molecular ion detected at *m*/*z* 1071.5776 [M+H]^+^.

### 2.3. Preparation of Dual-Prodrug Nanoparticles (DP–EP NPs)

DP–EP NPs were obtained by one-step nanoprecipitation. DOX-PRO and EXA-PRO were dissolved (EtOH) at a 1:2 molar ratio and mixed with DSPE-PEG_2k_ (drug:excipient = 10:1, *w*/*w*). The organic phase was rapidly added dropwise into deionized water under stirring, subsequent to organic solvent removal (nanoparticles were placed in dialysis bags with a molecular weight cutoff (MWCO) of 3500 for dialysis) and buffer exchange; suspensions were filtered (0.22 μm) and stored at 4 °C.

### 2.4. Physicochemical Characterization

The prepared nanoparticles were stored in dialysis bags with a molecular weight cutoff (MWCO) of 3500. After 24 h of dialysis, the free drugs diffused into the external solution. Subsequently, 100 μL of DP–EP NPs was precisely pipetted, diluted to 0.5 mL with dimethyl sulfoxide (DMSO), and the nanoparticle solution was ultrasonicated for 30 min to disrupt the nanoparticles to release prodrugs. The samples were then centrifuged at 12,000 rpm for 5 min. The supernatant was carefully collected, and the contents of DOX-PRO and EXA-PRO in the samples were detected by high-performance liquid chromatography (HPLC) and ultraviolet–visible spectroscopy (UV-Vis) in accordance with the aforementioned formulations (DL = 100 × (the weight of drug in NPs/the weight of NPs); EE = 100 × (the weight of drug in NPs/the weight of drug)).

### 2.5. Stability

After NPs were stored at 4 °C for 24 h or in serum-containing media (10% FBS in PBS; DMEM with 10% FBS) at 37 °C for 24 h, the particle size, polydispersity index (PDI), and zeta potential of the nanoparticles were measured using a Malvern Zetasizer Nano (Worcestershire, UK) series instrument.

### 2.6. In Vitro Hypoxia-Responsive Release of DOX and EXA

A dual-prodrug nano-system with a drug-to-excipient ratio of 10:1 (DOX PRO–EXA PRO: DSPE-PEG_2k_) was prepared with the DOX-PRO concentration of 0.5 mg/mL. Na_2_S_2_O_4_ was dissolved in a mixed solvent of acetonitrile: water (*v*:*v* = 1:1) to prepare release medium solutions with concentrations of 0 μM, 500 μM, 1 mM, and 5 mM. A total of 1 mL of the DP–EP NPs solution (with DOX-PRO concentration of 0.5 mg/mL) in a dialysis bag was immersed in 3 mL of the above-mentioned release media with different concentrations, respectively. At 37 °C, in a constant-temperature shaker, 500 μL of the release medium was sampled (an equal volume of fresh release medium was supplemented to the system to maintain a constant volume) at 2 h, 4 h, 6 h, 8 h, 10 h, and 24 h. The sampled solution was then sonicated for 10 min, and the concentration of DOX was determined by HPLC. The same operation method was applied to detect the concentration of EXA.

### 2.7. Cellular Culture

MCF-7, MCF-7/ADR cells were cultured in DMEM supplemented with 10% FBS and 1% penicillin/streptomycin at 37 °C, 5% CO_2_. The 4T1 cells were cultured in RPMI-1640 supplemented with 10% FBS and 1% penicillin/streptomycin at 37 °C (hypoxia, 1% O_2_ in a tri-gas incubator).

### 2.8. Cytotoxicity and Synergy Analysis

Using MCF-7 and 4T1 cells, the optimal synergistic concentration of the two prodrugs was determined by the results of cytotoxicity. The ratios of DOX-PRO and EXA-PRO were as follows: DOX-PRO at 10 μM; the concentrations of EXA-PRO were set to 40 μM (1:4), 20 μM (1:2), 10 μM (1:1), 5 μM (2:1), 2.5 μM (4:1), and 1.25 μM (8:1). After administration of DOX-PRO and EXA-PRO (physical mixture) at different ratios for 48 h, the cell viability was detected by CCK-8, and the IC_50_ value was calculated based on the viability. Compusyn software (1.2 version) was used to calculate the combination index (CI). The value of the CI reflects the synergistic effect of combination (CI < 1, a synergistic effect; CI = 1, an additive effect; CI > 1, an antagonistic effect).

### 2.9. Cellular Uptake

The 4T1 cells were seeded into a 24-well plate at a density of 5 × 10^4^ cells per well and cultured for an additional 24 h. After they adhered, the cells were treated with DOX, DOX-PRO + EXA-PRO, and DP–EP NPs (the concentration of 20 μM calculated by DOX). After further culturing for 2 h or 8 h in normoxic and hypoxic environments (normoxic environment: T = 37 °C, CO_2_ = 5.0%; hypoxic environment: T = 37 °C, CO_2_ = 5.0%, O_2_ = 2.5%), the medium was discarded, and the cells were washed three times with PBS, then fixed with 4% paraformaldehyde, stained with DAPI for nuclear labeling, and washed with PBS. Finally, observations were conducted using an inverted fluorescence microscope.

After incubation for 2 h and 8 h, the drug-containing culture medium was discarded, and the cells were washed three times with PBS. Then, 75 μL of triple-distilled water was added, and the cells were subjected to repeated freeze–thaw cycles between −80 °C and a 37 °C shaking incubator. The resulting cell aqueous solution was collected, mixed with 125 μL of acetonitrile by vortexing, and centrifuged at 12,000 rpm for 15 min. The supernatant was collected, and the concentrations of DOX and EXA were determined by HPLC-MS.

### 2.10. DNA Damage/Repair Readouts

The 4T1 cells were seeded into a 24-well plate. When the cell confluency reached up to 60–70%, the medium was discarded, and the cells were washed three times with PBS. The cells were treated with different formulations: DOX-PRO, EXA-PRO, DOX + EXA, and DP–EP NPs (the concentration of 20 μM calculated by DOX). After coincubation for 8 h in a hypoxic incubator, the cells were washed three times with PBS, then fixed with ice-cold methanol for 20 min. After washing with PBS three times, 0.2% Triton X-100 was added for permeabilization for 30 min. Then, after washing with PBS three times, the cells were blocked with 5% bovine serum albumin (BSA) at room temperature for 1 h. Subsequently, the cells were exposed to a mixture of primary antibodies (diluted at 1:500)—γ-H2AX (Histone H2AX monoclonal antibody) and 53BP1 (TP53BP1-Specific Polyclonal Antibody)—and incubated at 4 °C overnight. After washing with PBS three times, the cells were incubated with Alexa Fluor 488/594-labeled goat anti-mouse/rabbit secondary antibodies (diluted at 1:500), and incubated at 37 °C for 1 h in the dark. After washing with PBS three times, DAPI staining was performed at room temperature for 15 min. After imaging with an inverted fluorescence microscope, the average fluorescence intensity was analyzed using Image J software (classic version).

### 2.11. Immunogenic Cell Death Markers

#### 2.11.1. Cellular CRT Immunofluorescence

The 4T1 cells were seeded into a 24-well plate and incubated for 24 h to allow cell adhesion. The cells were divided into five groups: PBS control group, DOX-PRO group, EXA-PRO group, DOX + EXA group, and DP–EP NPs (dual-prodrug nanoparticles) group (the concentration of 10 μM calculated by DOX). The cells were cultured in a hypoxic incubator for 8 h, then transferred to a normoxic incubator for another 16 h. After washing three times with PBS, the cells were fixed with ice-cold methanol for 15 min. After removal of excess fixative, 2% bovine serum albumin (BSA) was used for blocking for 30 min. After blocking, Alexa Fluor^®^ 488 Anti-Calreticulin Antibody was administrated in the cells at 37 °C and incubated for 2 h. Finally, the cell nuclei were stained with DAPI for 15 min subsequent to washing cells with PBS three times. CRT-associated cellular fluorescence was imaged using an inverted fluorescence microscope and quantified using Image J.

#### 2.11.2. Intracellular HMGB1 Immunofluorescence

The 4T1 cells were seeded into 24-well plates and incubated for 24 h. The cells were treated with PBS, DOX-PRO, EXA-PRO, DOX + EXA, or DP–EP NPs at an equivalent DOX concentration of 20 μM. After incubation under hypoxia for 8 h followed by normoxia for 16 h, the cells were fixed with paraformaldehyde for 15 min and permeabilized with 0.1% Triton X-100 for 30 min. After blocking with 2% BSA for 30 min, the cells were incubated with the primary antibody (HMGB-1 Rabbit PAb) overnight at 4°C and subsequently with a CoraLite488-conjugated secondary antibody at 37°C for 2 h. Cell nuclei were stained with DAPI. Intracellular HMGB1 immunofluorescence was imaged using an inverted fluorescence microscope and quantified using Image J.

### 2.12. Evaluation of Chemoresistance Reversal in MCF-7/ADR Cells

MCF-7/ADR cells were seeded into a 24-well plate and cultured for 24 h. After discarding the medium, the cells were washed three times with PBS, then divided into three groups. DOX and DP–EP NPs (the concentration of 20 μM calculated by DOX) were administered to the treatment groups, while PBS was added in the control group. The cells were cultured in a hypoxic incubator for 2 h, 8 h in the dark. After incubation, the cells were washed with PBS three times, then fixed with 4% paraformaldehyde for 15 min, and stained with DAPI for 15 min. After washing, the results of uptake by the drug-resistant cells were observed under an inverted microscope.

A CCK-8 assay was used to investigate the resistance index (RI) and chemoresistance reversal effect of MCF-7/ADR cells. MCF-7/ADR cells were seeded into a 96-well plate at a density of 5 × 10^3^ cells per well and incubated for 24 h. Subsequently, the cells were treated with different concentrations of DOX formulations for 48 h. The values of IC_50_ were calculated to evaluate the reversal effect on multidrug resistance (MDR) based on parameters including the resistance index (RI) and reversal factor (RF). The formulas for calculating the RI and RF are as follows:

Resistance Index (RI) = IC_50(DOX-MCF-7/ADR)/_IC_50(DOX-MCF-7)_

Reversal Factor (RF) = IC_50(DOX-MCF-7/ADR)_/IC_50(DOX formulation-MCF-7/ADR)_

### 2.13. Pharmacokinetics in Rats

Fasted Sprague–Dawley (SD) rats were randomly divided into 2 groups with 4 rats per group. The rats were administered DOX + EXA solution or DP–EP NPs solution via the jugular vein, respectively. The dosage was equivalent to 2.5 mg/kg of DOX and 5 mg/kg of EXA. A total of 400 μL of blood was collected in a 1.5 mL tube pre-rinsed with heparin sodium from the jugular vein of each rat at 5 min, 15 min, 30 min, 45 min, 1 h, 2 h, 4 h, 6 h, 8 h, 12 h, 24 h, and 48 h. After centrifugation at 3500 rpm for 15 min, the plasma was separated and stored at −80 °C. A total of 50 μL of rat plasma was mixed with 10 μL of 10 mmol/L Na_2_S_2_O_4_ and vortexed for 3 min. After 10 min (the drug DOX or EXA was released from the prodrug), four volumes of acetonitrile containing the internal standard (Acetaminophen or Camptothecin) were added to the mixture to precipitate protein. After centrifugation at 12,000 rpm for 15 min, the collected supernatant was evaporated to dryness under a stream of nitrogen, and the residual was reconstituted in the mobile phase. Following a second centrifugation at 12,000 rpm for 15 min, an aliquot of 10 μL of the supernatant was injected in LC-MS/MS to detect the concentrations of DOX and EXA. The pharmacokinetic parameters of DOX and EXA in two groups were calculated using DAS 2.0 software.

Chromatographic and Mass Spectrometric Conditions

For DOX, chromatographic separation was performed on an ODS-BP column (2.1 mm × 150 mm, 5 μm). The mobile phase consisted of 0.1% formic acid in water and acetonitrile (70:30, *v*/*v*) at a flow rate of 250 μL/min. The column temperature was maintained at ambient conditions, and the injection volume was 10 μL. Mass spectrometric detection was carried out using an electrospray ionization (ESI) source in positive ion mode with multiple reaction monitoring (MRM). The ion spray voltage (IS) was set at 4500 V, curtain gas (CUR) at 25 psi, collision gas (CAD) at 10 psi, source temperature (TEM) at 600°C, ion source gas 1 (GSI) at 25 psi, and ion source gas 2 (GSII) at 35 psi. The MRM transitions were *m*/*z* 544.40 → 397.00 for doxorubicin and *m*/*z* 152.2 → 110.0 for Acetaminophen. The declustering potential (DP), entrance potential (EP), collision cell exit potential (CXP), and collision energy (CE) were set to 40 eV, 10 eV, 33 eV, and 14 eV, respectively.

For EXA, chromatographic separation was achieved on an ODS-BP column (2.1 mm × 150 mm, 5 μm). The mobile phase consisted of acetonitrile (containing 0.1% TFA) and water (containing 0.1% TFA) (70:30, *v*/*v*) at a flow rate of 300 μL/min. The column temperature was maintained at ambient conditions, and the injection volume was 10 μL. Mass spectrometric detection was performed using an electrospray ionization (ESI) source in positive ion mode with multiple reaction monitoring (MRM). The ion spray voltage (IS) was set at 5300 V, curtain gas (CUR) at 25 psi, source temperature (TEM) at 280 °C, collision gas (CAD) at 9 psi, ion source gas 1 (GS1) at 25 psi, and ion source gas 2 (GS2) at 30 psi. The MRM transitions were *m*/*z* 435.9 → 418.9 for exatecan (EXA) and *m*/*z* 349.1 → 305.3 for Camptothecin (CPT). The optimized compound parameters for EXA were as follows: declustering potential (DP) 70.0 eV, entrance potential (EP) 8.0 eV, collision energy (CE) 31.0 eV, and collision cell exit potential (CXP) 39.0 eV. For CPT, the optimized parameters were set to DP 70.0 eV, EP 10.0 eV, CE 26.0 eV, and CXP 20.0 eV.

### 2.14. Biodistribution in Tumor-Bearing Mice

Tumor-bearing mice were randomly divided into two groups: the IR808 group and the IR808/DP–EP NPs group. Mice in the two groups were injected with 100 μL of free IR808 and IR808/DP–EP NPs via the tail vein, respectively. The mice were anesthetized and imaged by an imaging system 0 h, 8 h, 12 h, 24 h, 48 h, and 72 h after administration (λ_Ex_ = 745 nm, λ_Em_ = 800 nm). The distribution of the two formulations in mice was assessed by the intensity of IR808 fluorescence. After 72 h, the mice were sacrificed, and their major internal organs and tumors were collected for ex vivo fluorescence imaging.

### 2.15. Antitumor Efficacy and Safety in Syngeneic 4T1 Tumor Model

The 4T1 cells were washed twice with PBS to remove dead cells. After digestion with trypsin for 2 min, culture medium was added to terminate the digestion. Following centrifugation at 1000 rpm for 5 min, the culture medium containing trypsin was discarded. In total, 2 mL of PBS was added to resuspend the cells. After centrifugation at 1000 rpm for 5 min, the supernatant was discarded and the residual cells were dispersed with PBS. The cell concentration was adjusted to 5 × 10^6^ cells/mL. A total of 100 μL of the diluted cell suspension was subcutaneously injected into the right axillary region of each mouse. After 7 days, when the tumors had grown to approximately 100 mm^3^, the syngeneic 4T1 tumor model was established successfully. Tumor-bearing mice were randomly divided into 5 groups: normal saline group, DOX-PRO group, EXA-PRO group, DOX + EXA group, and DP–EP NPs group. During the experiment, the mice had free access to food and water.

The experimental design is as follows:

(1) Normal saline group: Each mouse was injected with 200 μL of normal saline via the tail vein once every 2 days. (2) DOX-PRO group: The dosage was 2.5 mg/kg (calculated by DOX) via the tail vein once every 2 days. (3) EXA-PRO group: The dosage was 5 mg/kg (calculated by EXA) via the tail vein once every 2 days. (4) DOX + EXA group: It was administered at doses of 5 mg/kg EXA and 2.5 mg/kg DOX via the tail vein once every 2 days. (5) DP–EP NPs group: It was administered at doses of 5 mg/kg EXA-PRO (calculated by EXA) and 2.5 mg/kg DOX-PRO (calculated by DOX) via the tail vein once every 2 days.

Preparation of Intravenous Formulations:

DOX PRO solution: An amount of 10 mg of DOX PRO was accurately weighed and dissolved in 1 mL of DMSO. An aliquot of 100 μL of this solution was mixed with 400 μL of PEG400, followed by the slow, incremental addition of 500 μL of 10% Tween 80 in saline. The mixture was vortexed and sonicated in a water bath to obtain a final DOX PRO concentration of 1 mg/mL (equivalent to 0.5 mg/mL of DOX).

EXA PRO solution: EXA PRO (20 mg) was accurately weighed and dissolved in 1 mL of DMSO. A 100 μL aliquot was combined with 400 μL of PEG400, after which 500 μL of 10% Tween 80 in saline was added slowly in increments with vortexing and water bath sonication. The resulting EXA PRO concentration was 2 mg/mL (equivalent to 1 mg/mL of EXA).

EXA solution: EXA (10 mg) was accurately weighed and dissolved in 1 mL of DMSO. A 100 μL portion was taken and mixed with 400 μL of PEG400, followed by the slow, stepwise addition of 500 μL of saline. The mixture was vortexed and sonicated in a water bath to yield a final EXA concentration of 1 mg/mL.

DOX solution: DOX (5 mg) was accurately weighed and dissolved in 1 mL of DMSO. Subsequently, 100 μL of this solution was mixed with 400 μL of PEG400, and 500 μL of saline was added slowly in portions under vortexing and water bath sonication. The final concentration of DOX was 0.5 mg/mL.

DP-EP NPs formulation: DOX PRO (1 mg), EXA PRO (2 mg), and DSPE-PEG_2K_ (0.3 mg) were accurately weighed. The dual-prodrug nanoparticle preparation was carried out following the procedure described. After rotary evaporation, the formulation was reconstituted to a volume of 1 mL, yielding final concentrations of 2 mg/mL for EXA-PRO and 1 mg/mL for DOX-PRO.

Throughout the treatment period, tumor dimensions—length (a) and width (b)—were recorded daily with a Vernier caliper, while mouse body weights were simultaneously monitored. The tumor volume (v) was determined according to the formula: v = 0.5 × a × b^2^. On day 8, all animals were euthanized by cervical dislocation, after which major organs including the heart, liver, spleen, lungs and kidneys, as well as tumor tissues, were harvested. Blood samples were obtained via retro-orbital puncture and centrifuged at 3000 rpm for 15 min. The resulting supernatants (sera) were collected and analyzed using commercial kits for aspartate aminotransferase (AST), alanine aminotransferase (ALT), blood urea nitrogen (BUN), and creatinine (CREA) levels.

### 2.16. Statistics

The results of statistical analysis are expressed as mean ± standard deviation (SD). Statistical significance among three or more groups were determined using one-way ANOVA followed by Tukey’s multiple-comparison test. Time-dependent tumor-volume and body-weight data were analyzed using two-way repeated-measures ANOVA followed by Tukey’s test. A *p*-value < 0.05 was considered significant and a *p*-value < 0.01 was considered highly significant.

## 3. Results and Discussion

### 3.1. Synergy-Guided Ratio Selection

**Figure 2:**
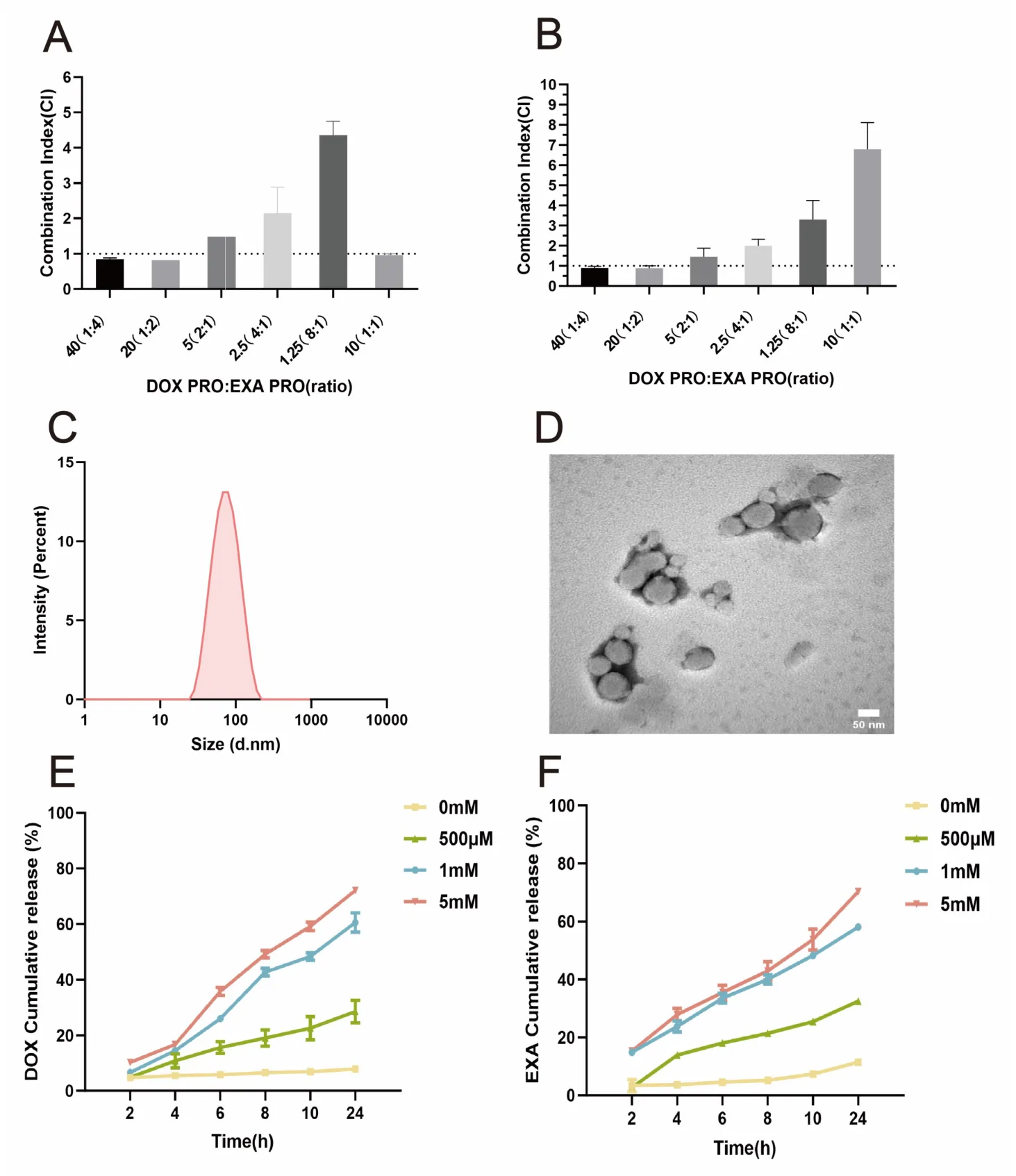
Physicochemical characterization and reductant-responsive release of DP-EP NPs. (**A**,**B**) Combination-index values for DOX-PRO/EXA-PRO mixtures in 4T1 and MCF-7 cells, respectively, *n* = 3; (**C**) hydrodynamic-size distribution; (**D**) TEM image (scale bar, 50 nm); and cumulative release of (**E**) DOX and (**F**) EXA in media containing 0–5 mM Na_2_S_2_O_4_ at 37 °C, *n* = 3. CI < 1 indicates a synergistic effect; CI = 1 indicates an additive effect; CI > 1 indicates an antagonistic effect.

Dose–response combination screening in MCF-7 and 4T1 cells (CCK-8, 48 h) established the synergy-optimized ratio for the two prodrugs before formulation (Figure 2A,B). Fixed-ratio combinations (DOX-PRO:EXA-PRO = 4:1, 2:1, 1:1, 1:2, and 1:4) were evaluated according to the Chou–Talalay combination index (CI). Across the clinically relevant effect window (Fa 0.5–0.9), the 1:2 ratio reproducibly generated synergism (CI = 0.88 in MCF-7; CI = 0.82 in 4T1), whereas DOX-enriched proportions drifted toward additivity or mild antagonism. This statistically anchored ratio was consequently fixed for all subsequent nanoassembly and in vivo dosing to preserve the pharmacodynamic complementarity of Topo II/Topo I inhibition while precluding the ratio erosion that undermines free-drug combinations.

Mechanistically, the observed synergy at a defined ratio is linked to the engagement of replication-associated DNA damage (Topo I) and interference with double-strand break repair (Topo II), which can limit compensatory repair and reduce the likelihood of cross-resistance. Selecting the 1:2 ratio a priori ensures that the nanoformulation is anchored to a pharmacologically validated proportion rather than a composition chosen solely by colloidal convenience.

### 3.2. Nanoassembly and Physicochemical Characterization of Dual-Prodrug Nanoparticles

Guided by combination-index screening, we fixed the DOX-PRO:EXA-PRO molar ratio at 1:2 and minimized excipient content with drug:DSPE-PEG_2k_ = 10:1, enabling one-step nanoprecipitation into stable co-assemblies. Dynamic light scattering (DLS) showed a hydrodynamic diameter of 75.70 ± 1.16 nm (Figure 2C), PDI 0.16 ± 0.01, and zeta potential −17.07 ± 1.25 mV, while TEM revealed uniform, near-spherical morphology without visible aggregation (Figure 2D). The drug-as-carrier strategy yielded high effective loadings by design, with DOX-PRO achieving a drug loading (DL) of 62.95% (encapsulation efficiency, EE, 81.88%) and EXA-PRO attaining a DL of 81.14% (EE 93.31%) (Table S1). Both prodrugs, sharing the same squalene–azo hydrophobic motif, were co-dissolved before nanoprecipitation and were both detected in the purified nanoparticles. DLS showed a single narrow size distribution (low PDI), and TEM revealed uniform particles without phase separation. These results confirm that DOX-PRO and EXA-PRO co-assemble into a single nanoparticle population rather than forming two separate populations. The hydrodynamic diameter of approximately 76 nm falls within the canonical range compatible with the enhanced permeability and retention (EPR) effect, while the mildly negative surface charge, in conjunction with PEGylated coronas, promotes serum stability and reduces opsonization.

From a translational perspective, anchoring the co-assembly at a 1:2 molar ratio is designed to preserve the synergy-optimized drug proportion both systemically and at the tumor site, thereby circumventing the pharmacokinetic (PK) mismatch inherent to free-drug combinations. The 10:1 drug-to-PEG-lipid formulation strikes a balance between colloidal stability and a minimal inert excipient burden, facilitating both dose intensification and manufacturability. Short-term colloidal stability in 10% FBS/PBS and DMEM at 37 °C and during 4 °C storage showed negligible size drift or polydispersity index (PDI) broadening (Figure S5), supporting robustness for subsequent in vitro and in vivo studies.

### 3.3. Hypoxia-Triggered Co-Release of DOX and EXA Under Reductive Conditions

Under hypoxia-mimicking conditions, the dual-prodrug nanoparticles exhibited synchronized and markedly accelerated release of both payloads relative to normoxic control (Figure 2E,F). At 1 mM Na_2_S_2_O_4_, 24 h cumulative release reached ~64.8% for DOX and ~57.9% for EXA, compared with ~7.9% and ~11.4% at 0 mM Na_2_S_2_O_4_; increasing the reductant (e.g., 1–5 mM Na_2_S_2_O_4_) further elevated release with convergent kinetics between the two drugs. This convergence indicates that the azo-linker cleavage is the common rate-limiting step, implying that co-delivery avoids any payload-specific kinetic delay that could otherwise disrupt the temporal coordination of Topo II/I targeting under hypoxia. In contrast, in normoxic media both payloads remained largely retained, indicating low leakage from the squalene-anchored core and supporting the premise that matrix integrity is maintained until trigger activation.

Under hypoxia-mimicking conditions simulated by reducing agents, release data indicate that the prodrug nanoparticles trigger cleavage of the N=N bond under hypoxic conditions, resulting in the release of the parent drug, while the PEGylated corona and slightly negative zeta potential stabilize the colloid and prevent premature unloading. In practice, such on-demand co-release is expected to enhance pharmacological synergy by aligning the exposure windows of DOX and EXA within the hypoxic regions of tumors, thereby protecting normal tissues from the adverse effects of conventional chemotherapy. It should be noted that Na_2_S_2_O_4_ is a chemical surrogate; while standard for in vitro modeling, its redox profile does not recapitulate the full spectrum of enzymatic azoreductases and intracellular reductants. In hypoxic cells, azo-bond reduction is governed by NAD(P)H-dependent reductases, competition between oxygen and the prodrug for reducing equivalents, enzyme abundance, intracellular localization, and the heterogeneous oxygen and redox gradients within tumors. Therefore, the release rates measured in the Na_2_S_2_O_4_ system should be regarded as evidence of chemical cleavage competence and as an approximate upper-bound response rather than quantitative predictions of in vivo release kinetics or hypoxia selectivity. Accordingly, we interpret these results as upper-bound responsiveness, to be complemented by cellular assays under controlled hypoxia and, ultimately, by in vivo readouts where the heterogeneous oxygen landscape and reductase expression are operative. Stability assays (Figure S5) showing minimal size/PDI drift across serum-containing media further argue that the observed release arises from triggered linker cleavage rather than colloidal breakup.

### 3.4. Cytotoxicity, Cellular Uptake, DNA-Damage/Repair Interference and Immunogenic Cell Death

The IC_50_ values of free drugs and formulations in 4T1 cells are shown in Figure S6. Following 72 h treatment, time-dependent cytotoxicity enhancement was observed for all prodrug formulations compared to 48 h. IC_50_ values decreased approximately 3-fold for DOX PRO (21.63 ± 2.47 μM vs. 66.37 ± 4.08 μM) and 6-fold for EXA PRO (12.51 ± 0.61 μM vs. 81.17 ± 4.59 μM). Remarkably, DP-EP NPs achieved IC_50_ values at 72 h comparable to those of free DOX (3.59 ± 0.1 μM DOX-equivalent) and free EXA (7.18 ± 0.2 μM EXA-equivalent). This indicates nanoparticle internalization via endocytosis, efficient cellular fusion, and sustained drug release within the tumor microenvironment, ultimately yielding cytotoxicity equivalent to free drugs. For MCF-7 cells, the cytotoxicity trend at 48 h was consistent with that observed in 4T1 cells. However, by 72 h, no significant differences in cytotoxicity were detected among treatment groups. This outcome likely reflects cell line-specific variations in uptake capacity and hypoxia-dependent drug release kinetics. Therefore, subsequent cellular uptake studies were conducted using 4T1 cells for further mechanistic investigation.

**Figure 3:**
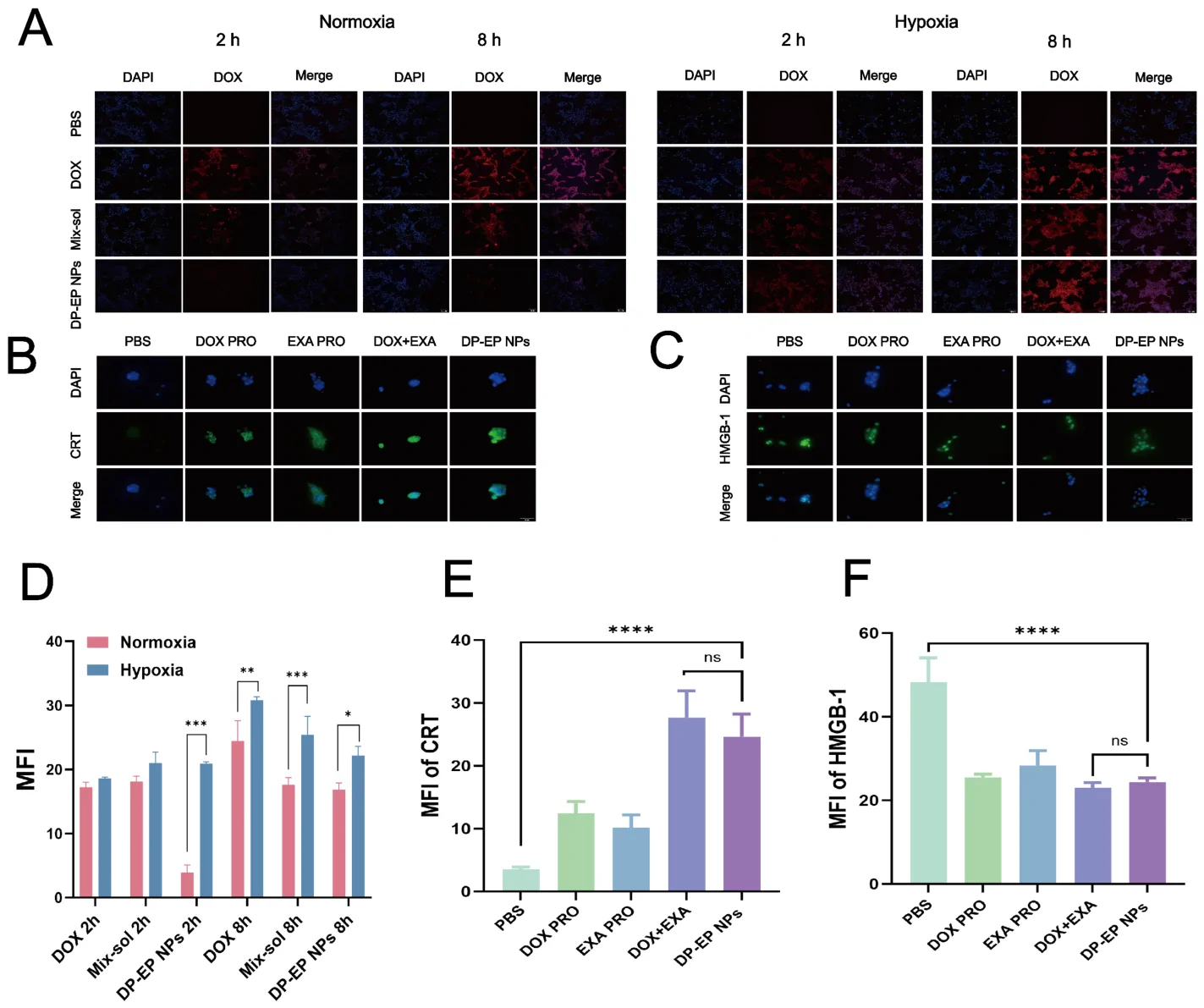
Cellular DOX fluorescence and immunogenic-cell-death-associated markers. (**A**) Fluorescence images of 4T1 cells treated with DOX, a DOX-PRO/EXA-PRO mixture, or DP-EP NPs for 2 or 8 h under normoxia or hypoxia (scale bar, 100 μm; *n* = 3); (**B**) cellular CRT immunofluorescence (scale bar, 50 μm; *n* = 3); (**C**) intracellular HMGB1 immunofluorescence (scale bar, 50 μm; n = 3); and quantitative analysis of (**D**) DOX fluorescence, (**E**) CRT-associated cellular fluorescence, and (**F**) intracellular HMGB1 fluorescence, *n* = 3. *, *p* < 0.05; **, *p* < 0.01; ***, *p* < 0.001; ****, *p* < 0.0001.

Across hypoxic 4T1 cells, the dual-prodrug nanoparticles (DP-EP NPs) produced comparable intracellular accumulation to that of the free-drug mixture or single-prodrug controls, whereas under normoxic conditions, it was significantly lower (Figure 3A,D). The inverted fluorescence microscope (IFM) showed bright cytoplasmic puncta transitioning to a more diffuse signal over time under hypoxia conditions, consistent with endocytic entry followed by trigger-facilitated payload DOX release. And within 2 h, there was no significant difference in the uptake capacity of 4T1 cells between the DOX group and the Mix-sol group under both normoxic and hypoxic conditions. In contrast, the DP-EP NPs group exhibited significantly higher DOX cellular uptake under hypoxic conditions compared to normoxic conditions. Meanwhile, in the 8 h uptake assay, the uptake trends of the three different administration groups were generally consistent with those observed at 2 h, yet the free DOX group expression level remained the highest. The underlying reason might be that after formulating DOX and EXA into hypoxia-sensitive prodrugs with cleavable bonds, the drug release might lead to delayed expression. Furthermore, the nanoparticles formed by self-assembly of DOX PRO and EXA PRO are likely taken up by cells via endocytosis, which prolongs the intracellular release of DOX, thereby potentially reducing its efficacy in entering the nucleus to damage DNA. This observation is consistent with the results of our Cytotoxicity Assay (Figure S6). In summary, the drug uptake at 8 h was higher in all treatment groups compared to the corresponding groups at 2 h, and nanoparticles were more readily internalized under hypoxic than normoxic conditions, leading to enhanced release of the parent drug. These findings suggest that cellular drug uptake is both time-dependent and hypoxia-sensitive. The uptake advantage persisted when the 1:2 (DOX-PRO:EXA-PRO) molar ratio was varied modestly around the optimum, suggesting that colloidal features and the hypoxia-labile linker—not only the loading ratio—contribute to cell-level delivery efficiency. The increased intracellular DOX-associated fluorescence and downstream pharmacological responses observed at 2.5% O_2_ are consistent with activation of the formulation in a biologically hypoxic environment, but they do not directly identify or quantify azo-bond cleavage. Further studies using NADPH-dependent azoreductase, hypoxic cell lysates, or microsomal reductase systems combined with direct HPLC or LC-MS measurement of released DOX and EXA will be required to define the enzymatic activation kinetics and oxygen dependence of the dual-prodrug system.

To evaluate the hypoxia-responsive drug release behavior of the prodrug nanoparticles, 4T1 cells were incubated with the nanoparticles under normoxic or hypoxic conditions for 8 h, followed by fluorescence microscopy imaging and LC/MS quantification. As shown in Figure 3A, under normoxic conditions, no DOX fluorescence was detected within the cells after 8 h of incubation, indicating that the prodrug itself is non-fluorescent and that the azo bond remains stable and intact under normal oxygen tension. In contrast, under hypoxic conditions, strong DOX fluorescence was observed in the cells, confirming the cleavage of the azo bond and the subsequent release of DOX. Given the structural similarity of the prodrugs (DOX-based and EXA-based), it is reasonable to infer that EXA would undergo a similar azo-bond cleavage and release pattern under the same conditions. To further corroborate this observation, we performed quantitative LC/MS analysis of intracellular drug concentrations after 8 h of incubation. As depicted in Figure S7, the azo bond remained largely intact under normoxia, with negligible release of either DOX or EXA. Under hypoxia, however, the intracellular concentrations of DOX and EXA reached approximately 230 ng/mL and 254 ng/mL, respectively, confirming efficient hypoxia-triggered drug release at the cellular level.

Tumor hypoxia is determined by the dynamic balance between oxygen delivery and cellular oxygen consumption. Rapid mitochondrial respiration in proliferating tumor cells, together with inadequate perfusion caused by abnormal tumor vasculature, progressively lowers the local oxygen tension. Importantly, hypoxia itself should not be considered equivalent to a chemically reducing environment. Rather, reduced oxygen availability favors bioreductive activation by decreasing competition from molecular oxygen for electrons supplied by NAD(P)H-dependent reductases, thereby facilitating azo-bond reduction and subsequent regeneration of DOX and EXA. The cellular respiration rate therefore acts as an upstream determinant of local oxygen availability, whereas the actual cleavage reaction depends on reductase activity and the availability of reducing cofactors. Tumor hypoxia can be characterized by oxygen tension and complementary indicators such as HIF-1α stabilization, pimonidazole-adduct formation, and CAIX expression, while the NAD(P)H/NAD(P)^+^ balance and reductase activity more directly reflect the capacity for azo-bond bioreduction. These factors collectively determine the biological environment in which the dual prodrugs are expected to undergo activation.

Calreticulin (CRT) and HMGB1 were selected because they are two canonical and functionally complementary hallmarks of immunogenic cell death. CRT exposure represents an early “eat-me” signal that promotes the recognition and phagocytic uptake of dying tumor cells by antigen-presenting cells, whereas HMGB1 release occurs at a later stage and functions as a danger signal that promotes innate immune activation through receptors such as TLR4 and RAGE. Thus, the combined assessment of CRT and HMGB1 covers two distinct stages of the ICD process: tumor-cell recognition and subsequent immune stimulation. Importantly, DP-EP NPs induced ICD-associated molecular changes under hypoxic conditions, characterized by increased CRT-associated cellular fluorescence and decreased intracellular HMGB1 fluorescence (Figure 3B,C,E,F). These changes were more pronounced than those induced by the individual prodrugs and were comparable to those observed following treatment with the DOX + EXA mixture. The combined changes in CRT and intracellular HMGB1 are consistent with the redistribution of representative damage-associated molecular patterns following treatment with DP–EP NPs. Such ICD signatures provide a mechanistic bridge to in vivo efficacy by potentially priming antitumor immunity in addition to direct cytotoxicity [31,32]. In the MCF-7/ADR cell line, DP-EP NPs increased nuclear DOX signals and partially restored sensitivity (Figure S8, Table S2), consistent with endocytic bypass of efflux and on-site regeneration of active drugs after hypoxia-triggered cleavage. This finding further suggests that DP-EP NPs overcome resistance primarily through nanoparticle-mediated cellular entry, enhanced intracellular drug retention, and complementary Topo I/II inhibition, rather than through direct suppression or regulation of efflux-transporter expression.

Together, these cellular data build a coherent picture: hypoxia-responsive co-delivery improves intracellular exposure, synchronizes DNA-damage pathways, and adds an ICD component, establishing a multi-axis rationale for the superior antitumor outcomes observed later in vivo.

**Figure 4:**
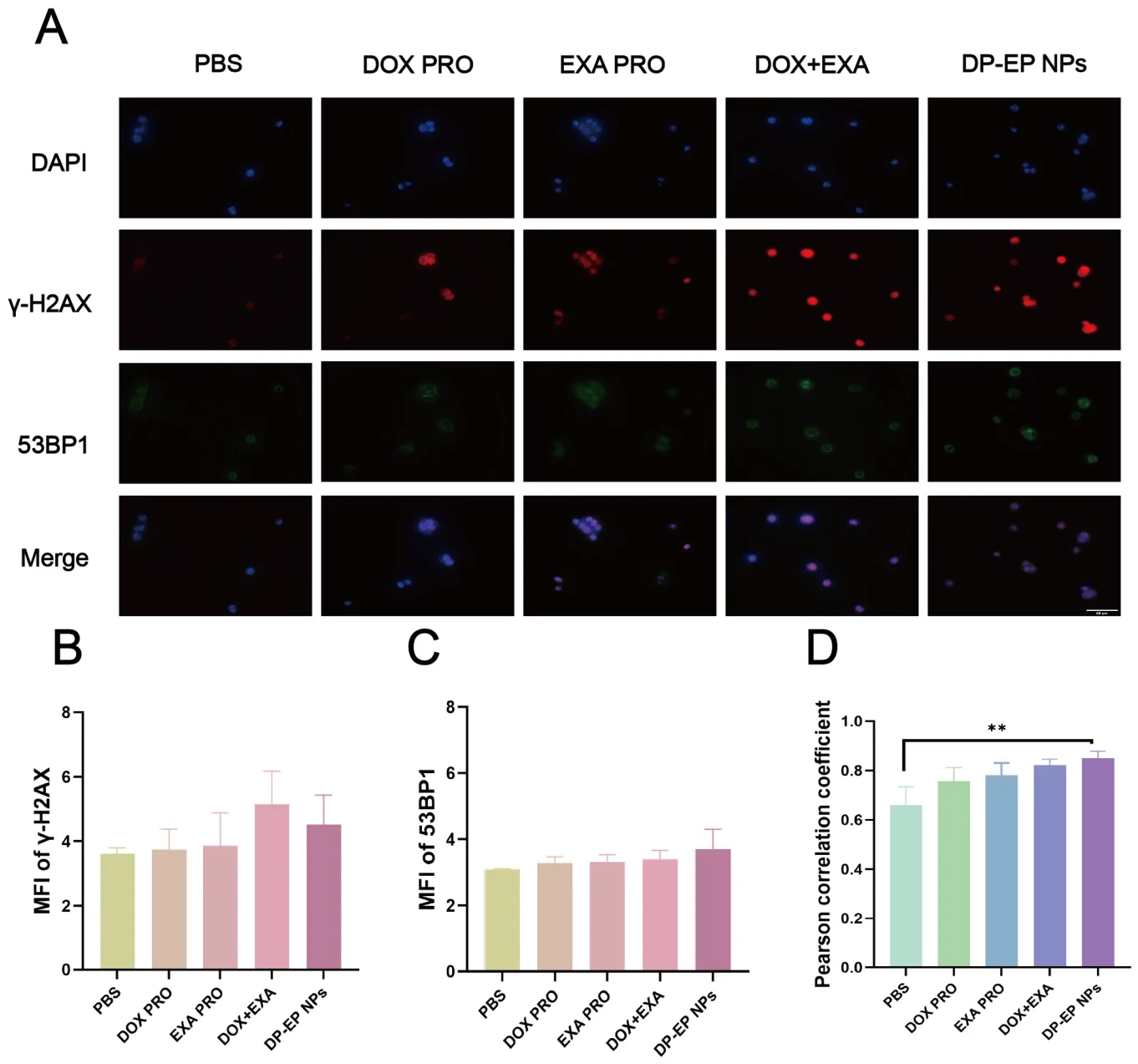
DNA-damage signaling in 4T1 cells under hypoxia. (**A**) Immunofluorescence images of γ-H2AX and 53BP1 (scale bar, 50 μm; *n* = 3); (**B**,**C**) mean fluorescence intensities of γ-H2AX and 53BP1, *n* = 3; and (**D**) Pearson colocalization coefficient for γ-H2AX and 53BP1, *n* = 3. **, *p* < 0.01.

Concordant with enhanced intracellular delivery, DP-EP NPs elicited robust DNA-damage signaling relative to comparators (Figure 4A–D). Hypoxia (modeled as in Figure 4A) increased the number and intensity of γ-H2AX foci and sharpened 53BP1 colocalization, indicating efficient double-strand break formation and recruitment of repair scaffolds [33]. Single-prodrug NPs activated these markers to a lesser degree and with asynchronous kinetics, whereas the dual formulation produced coincident peaks in γ-H2AX and 53BP1, aligning with the premise that synchronized Topo II (DOX) and Topo I (EXA) pressure overwhelms compensatory repair. It should be noted that the increased γ-H2AX and 53BP1 signals, together with their nuclear spatial association, support enhanced DNA-damage signaling following DP–EP NP treatment. However, these findings do not directly establish inhibition of DNA-repair activity, because the abundance and persistence of DNA-damage-associated foci may reflect both increased damage formation and ongoing recruitment of repair machinery. Cell-cycle analysis (Figure S9) corroborated this division of labor—DOX biasing G2/M arrest, EXA biasing S-phase interference—with the dual nanoparticles showing a bimodal accumulation that reflects concurrent pathway engagement.

### 3.5. Pharmacokinetics and Biodistribution

**Figure 5:**
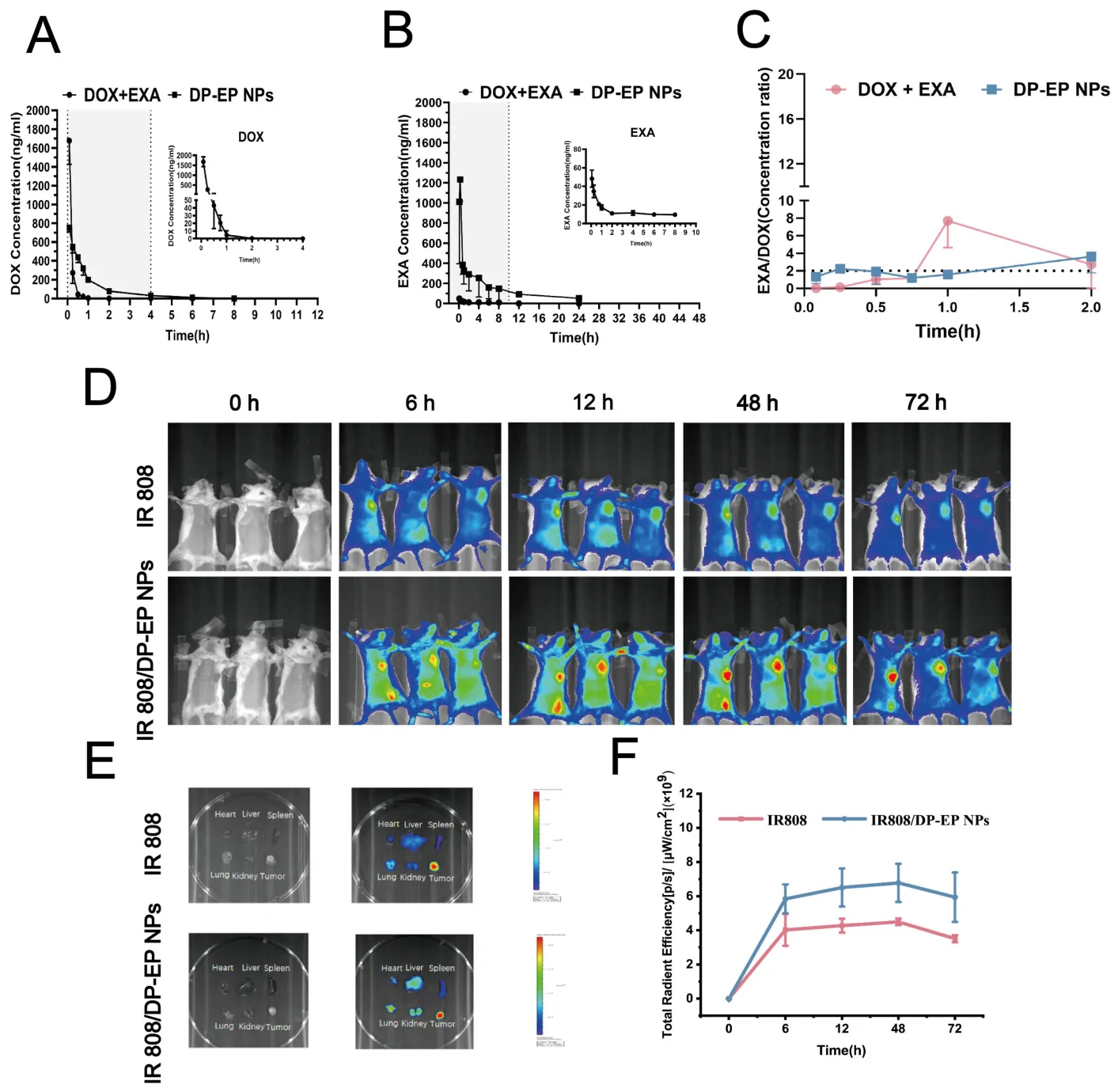
Pharmacokinetics and fluorescence distribution. Plasma concentration-time profiles of total releasable (**A**) DOX and (**B**) EXA equivalents after administration of DOX + EXA or DP-EP NPs, *n* = 4; (**C**) plasma EXA/DOX concentration ratio during the first 2 h, *n* = 4; (**D**) serial whole-body fluorescence images of free IR808 and IR808-labeled DP-EP NPs; (**E**) ex vivo fluorescence images of tumors and major organs at 72 h; and (**F**) quantitative tumor fluorescence. The small graphs in subfigures (**A**,**B**) represent the DOX or EXA blood drug concentration graphs of DOX + EXA group during the period when the shaded area represents the time.

In rats, DP-EP NPs substantially prolonged systemic exposure of both drugs compared with the free-drug mixture (Figure 5A,B, Table 1). For doxorubicin, the nanoparticles increased t½ by ~9-fold, AUC by ~1.8-fold, and MRT by ~10.2-fold, while reducing plasma clearance accordingly; for exatecan, AUC increased by ~47.0-fold and MRT ~6.9-fold, accompanied by a pronounced decrease in clearance. To investigate whether the nanoformulation co-loaded with the two drugs releases the drugs at a constant ratio consistent with the calculated drug synergy index after entering the systemic circulation, we further explored the concentration ratio of EXA/DOX within 2 h. As shown in Figure 5C, the EXA/DOX ratio in the co-loaded nanoparticle group could be maintained at the preset ratio within 2 h, whereas the ratio in the physically mixed group of the two free drugs fluctuated significantly, deviating from the initial ratio of 2:1. When DOX and EXA were administered as a free-drug mixture, their distinct distribution and elimination profiles caused rapid deviation from the initially administered ratio, reducing the probability that both drugs reached the tumor at pharmacologically complementary concentrations. In DP-EP NPs, the two prodrugs were incorporated into the same nanoscale assembly and consequently shared a common transport pathway during circulation. The maintenance of the plasma EXA/DOX ratio during the early circulation phase demonstrated that co-assembly effectively reduced differential pharmacokinetic drift between the two agents. Concentration–time curves displayed flattened early peaks and sustained plateaus, a pattern favorable for minimizing acute toxicity while maintaining cytotoxic pressure over time. These PK shifts are attributable to PEG-assisted stealth and the squalene-anchored core, which together mitigate rapid renal/hepatic elimination and curtail premature leakage.

Nanoparticle-associated fluorescence distribution analyses corroborated the PK findings. Ex vivo organ readouts revealed enhanced tumor accumulation and prolonged retention for DP-EP NPs relative to the free drug, with reduced off-target exposure in dose-limiting tissues (Figure 5D,F). The free IR808 group showed fluorescence accumulation at the tumor site, indicating that free IR808 itself possesses inherent tumor-targeting ability, and the fluorescence gradually diminished after 48 h. For the DP-EP NPs group, obvious fluorescence accumulation at the tumor site was observed as early as 6 h, with the fluorescence intensity significantly higher than that of the free IR808 group. Moreover, the fluorescence intensity reached its peak at 48 h, indicating the maximum fluorescence accumulation at this time point; subsequently, the drug began to be metabolized and eliminated from the body, leading to a decrease in fluorescence intensity. In vivo imaging results demonstrated that after encapsulating the drugs into nanoformulations, the passive enhanced permeability and retention (EPR) effect enables targeted antitumor efficacy, significantly prolongs the in vivo retention time of the drugs, and thereby further exerts a more potent therapeutic effect. The net effect is an improved tumor-to-normal tissue exposure ratio, consistent with the EPR-compatible particle size (approximately 76 nm) and colloidal stability documented earlier. Mechanistically, sustained intratumoral presence provides a temporal window in which hypoxia-triggered linker scission can operate, aligning drug activation with microenvironmental cues. In summary, the stronger and more persistent tumor-associated fluorescence of IR808-labeled DP-EP NPs supports enhanced tumor accumulation and retention of the nanoassembly. The LC-MS/MS pharmacokinetic analysis provides complementary direct quantification of DOX and EXA equivalents in plasma, whereas the fluorescence study provides spatial and temporal information regarding nanoparticle-associated tumor localization.

### 3.6. In Vivo Antitumor Efficacy

**Figure 6:**
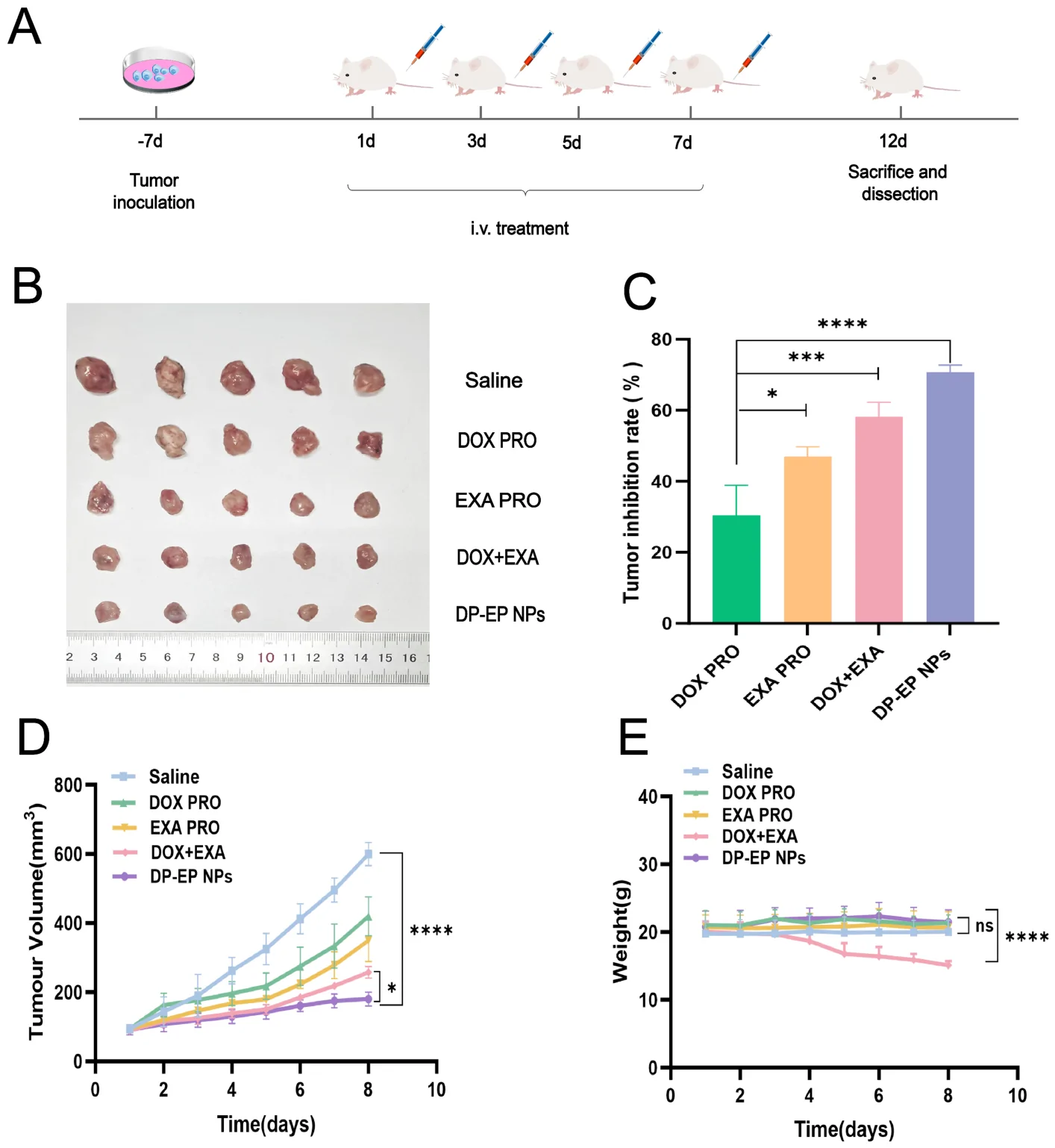
Antitumor efficacy in 4T1 tumor-bearing BALB/c mice (*n* = 5). (**A**) Tumor-inoculation and treatment schedule; (**B**) excised tumors at the study endpoint; (**C**) tumor-inhibition rate; (**D**) tumor-growth curves; and (**E**) body-weight changes during treatment. *, *p* < 0.05; ***, *p* < 0.001; ****, *p* < 0.0001.

In 4T1 tumor-bearing mice, DP-EP NPs exhibited the most pronounced tumor growth delay and the lowest terminal tumor weights, showing statistically significant differences relative to the saline, free-drug combination (DOX + EXA), and individual prodrug (DOX-PRO, EXA-PRO) control groups (Figure 6A–D). The tumor volume curves demonstrated that the terminal tumor volumes across groups followed the order DP-EP NPs < DOX + EXA < EXA-PRO < DOX-PRO < saline, indicating that the DP-EP NPs group possessed the strongest tumor-inhibitory efficacy. This finding is consistent with the previously observed synergistic effects at the cellular level and the pharmacokinetic/biodistribution (PK/BD) advantages. These outcomes are consistent with a mechanism in which prolonged circulation and tumor accumulation preserve a synergy-optimized 1:2 exposure, while hypoxia-triggered co-release synchronizes Topo II/I engagement at the disease site.

The superior antitumor activity of DP-EP NPs can be attributed to the integration of three complementary features: coordinated systemic exposure of DOX and EXA, reductive activation of the azo-linked prodrugs under hypoxia-mimicking conditions, and induction of ICD-associated cellular responses. Co-assembly stabilized the predefined synergistic ratio during the early circulation phase, whereas hypoxia-responsive cleavage supported concurrent payload release after cellular delivery. In parallel, increased calreticulin-associated fluorescence and reduced intracellular HMGB1 staining suggested the generation of immunologically relevant danger signals (Figure 3B,C,E,F). This combination of exposure coordination, microenvironment-responsive activation, and ICD-associated signaling provides a mechanistic basis for the improved efficacy of DP-EP NPs over the dose-equivalent free-drug combination. It should be pointed out that for further translation, the drug ratio should be optimized using human-relevant PK/PD data, together with direct tissue drug quantification, long-term safety evaluation, batch reproducibility, and formulation stability studies.

### 3.7. In Vivo Tolerability

As shown in Figure 6E, mice in the DOX + EXA group exhibited a decrease in body weight during the treatment period, whereas no significant changes in body weight were observed in the other groups, suggesting that the physical mixture of DOX and EXA may have higher toxicity. According to Figure S10, renal function assessment based on creatinine (CREA) levels indicated varying degrees of impairment across all treatment groups; however, all values remained within an acceptable range, and no obvious abnormalities in liver and kidney function were detected in the other treatment groups. H&E staining revealed evident renal injury across treatment groups, characterized by tubular damage including brush border loss in proximal tubules, cytoplasmic vacuolization, and cast formation (damaged areas circled in black). Notably, these alterations remained within acceptable limits. In tumor tissues, the DP-EP NPs group exhibited the most extensive necrotic area, indicating superior antitumor efficacy. Mild cardiac and hepatic damage was observed exclusively in the free DOX + EXA combination group, while no significant abnormalities were detected in other treatment groups. Spleen and lung sections showed no apparent histological changes across all groups. Taken together with the flattened early plasma peaks and sustained plateaus in pharmacokinetics, these data suggest mitigation of acute toxicity typically associated with anthracycline-containing regimens.

## 4. Conclusions

The reductant-responsive squalene-based dual-prodrug nanoparticles, co-delivering doxorubicin prodrug and exatecan prodrug at a predefined synergistic ratio of 1:2, were successfully developed. These nanoparticles were constructed via a minimalist drug-as-carrier strategy, forming uniform PEG-stabilized particles approximately 76 nm in size. This platform exhibited high drug loading, low premature leakage under normoxic conditions, and synchronous hypoxia-activated drug release. In various model systems, the nanoparticles significantly enhanced cellular uptake, amplified DNA-damage response (as demonstrated by elevated γ-H2AX and 53BP1 signals), induced ICD-associated molecular changes, prolonged systemic circulation, and promoted tumor-selective drug accumulation. Compared to the equivalent dose of the free-drug combination, the dual-prodrug nanoparticles demonstrated significantly improved antitumor efficacy and enhanced systemic tolerability. By achieving synchronized pharmacokinetics and pharmacodynamics through systemic maintenance of the synergistic drug ratio and tumor-hypoxia-specific activation via an azo-based linker, this excipient-minimal design provides a scalable and promising strategy for combination chemotherapy and can be readily adapted for other rationally designed drug pairs.

## Figures and Tables

**Table 1:** Pharmacokinetic parameters of DOX + EXA and DP-EP NPs (*n* = 4).

Pharmacokinetic Parameters	EXA	EXA (DP-EP NPs)	DOX	DOX (DP-EP NPs)
AUC_(0-t)_ (mg/L·h)	0.107 ± 0.045	5.031 ± 0.928	0.412 ± 0.054	0.745 ± 0.022
MRT_(0-t)_ (h)	2.015 ± 0.477	13.942 ± 1.09	0.100 ± 0.020	1.019 ± 0.133
CL_Z_ (L/h/kg)	29.681 ± 9.426	0.721 ± 0.143	6.129 ± 0.811	3.299 ± 0.135
t _1/2z_ (h)	5.625 ± 2.008	27.682 ± 9.246	0.119 ± 0.410	1.072 ± 0.244

## Data Availability

The data generated in this study are available from the corresponding author on request.

## Supplementary Materials

The following supporting information can be downloaded at: https://www.mdpi.com/article/10.3390/pharmaceutics18091155/s1, Table S1. Encapsulation Efficiency and Drug Loading Capacity of the Dual Prodrug in Nanoparticles. Table S2. RI and RF Values of MCF-7 and MCF-7/Adr Cells Treated with Different DOX Formulations at 48 h (n = 3). Figure S1. The ^1^H NMR spectrum of DOX-PRO. Figure S2. The MS spectrum of DOX-PRO. Figure S3. The ^1^H NMR spectrum of EXA-PRO. Figure S4. The MS spectrum of EXA-PRO. Figure S5. Stability of DP-EP NPs in Different Media. (A) Changes in particle size and PDI over 7 days at 4°C; (B) Changes in particle size and PDI within 24 h in PBS containing 10% FBS at 37°C; (C) Changes in particle size and PDI within 24 h in DMEM at 37°C. Figure S6. IC_50_ Values of Different Administration Groups on 4T1 (A) and MCF-7 (B) Cells at Different Time Points. Figure S7. Intracellular release of DOX and EXA from prodrug nanoparticles under normoxia and hypoxia. Figure S8. (A) Doxorubicin (DOX) Uptake Under Hypoxic Conditions (MCF-7/ADR Cells); (B) Fluorescence Quantitative Results by Image J; (C) MCF-7/ADR Cell Viability After Administration in Different Treatment Groups (72 h). Figure S9. Different Administration Groups of 4T1 Cells. (A) Cell Cycle Arrest Profiles; (B) Stacked Histogram of Cell Cycle Distribution Percentages. Figure S10. Tolerability evaluation. (A) H&E-stained sections of major organs and tumors (scale bar, 50 μm); and (B) serum biochemical indices of liver and kidney function.

## Abbreviations

EXA	Exatecan
EXA PRO	Exatecan Prodrug
SQ	Squalene
DOX	Doxorubicin
DOX PRO	Doxorubicin Prodrug
DP-EP NPs	Doxorubicin/Exatecan Prodrug Nanoparticles
