# Supplementary material for: Reductant-Responsive Squalene Dual-Prodrug Nanoparticles Co-Delivering Doxorubicin and Exatecan for Synergistic Breast Tumor Therapy
Source: Pharmaceutics. 2026 Sep 15;18(9):1155. doi: 10.3390/pharmaceutics18091155 (PMC13610880; doi:10.3390/pharmaceutics18091155)
Supplement: Supplementary file 1 [file pharmaceutics-18-01155-s001.zip › pharmaceutics-4494159-supplementary.pdf]

Hypoxia-responsive squalene dual-prodrug nanoparticles co-delivering doxorubicin and exatecan for synergistic breast tumor therapy

Li Wang [a,1], Ning Yang [a,1], Yina Sa [a], Zhenghua Li [a], Yanlong Qiao [a], Yanxin Yang [a], Lixue Chen [a], Xiangyu Liu [*,b] and Lei Li [*,a]

[a] *College of Pharmacy, Dalian Medical University, Dalian, 116044, PR China.*

[b] *Department of Day Surgery, The First Affiliated Hospital of Dalian Medical University, Dalian 116021, PR China.*

[1] *These authors contributed equally.*

[*] *Corresponding author.*

E-mail address: liuxiangyu@firsthosp-dmu.com (X. Liu); lilei0332@126.com (L. Li).

1. Tables

Table S1. Encapsulation Efficiency and Drug Loading Capacity of the Dual Prodrug in Nanoparticles.[a]

Dual Prodrug in Nanoparticles	DL (%)	EE（%）
**DOX-PRO**	60±0.05	81.88±0.06
**EXA-PRO**	81.14±0.01	93.31±0.01

a: The values of DL and EE were measured independently, n=3.

Table S2. RI and RF Values of MCF-7 and MCF-7/Adr Cells Treated with Different DOX Formulations at 48 h (n=3)

DOX Formulation	IC_{50}（μM）		RI	RF
	MCF-7/ADR	MCF-7		
**DOX**	19.60±0.12	0.38±0.08	52	-
**DP-EP NPs**	34.31±2.7	22.29±2.27	1.54	3.3

2. Figures

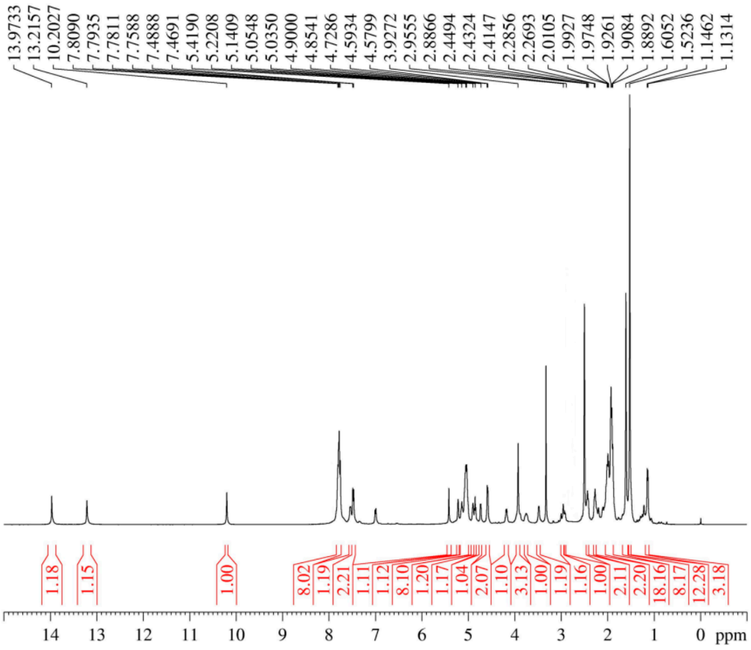

Figure S1. The ^{1}H NMR spectrum of DOX-PRO.

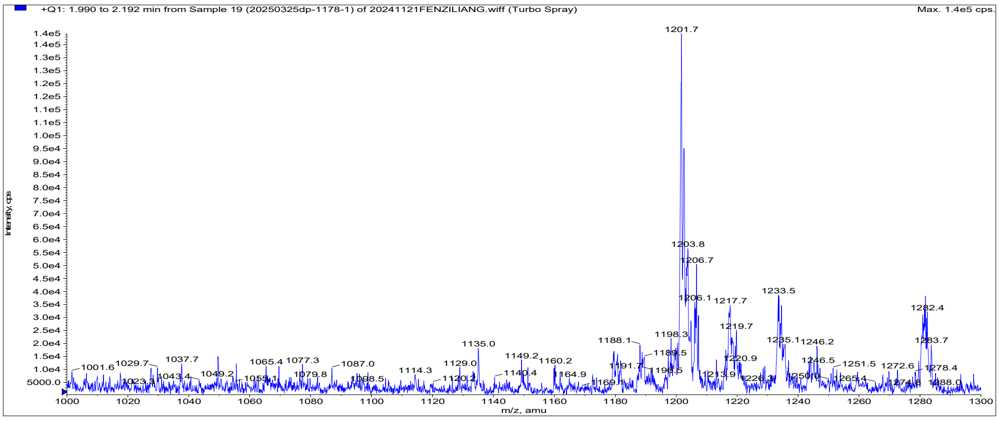

Figure S2. The MS spectrum of DOX-PRO.

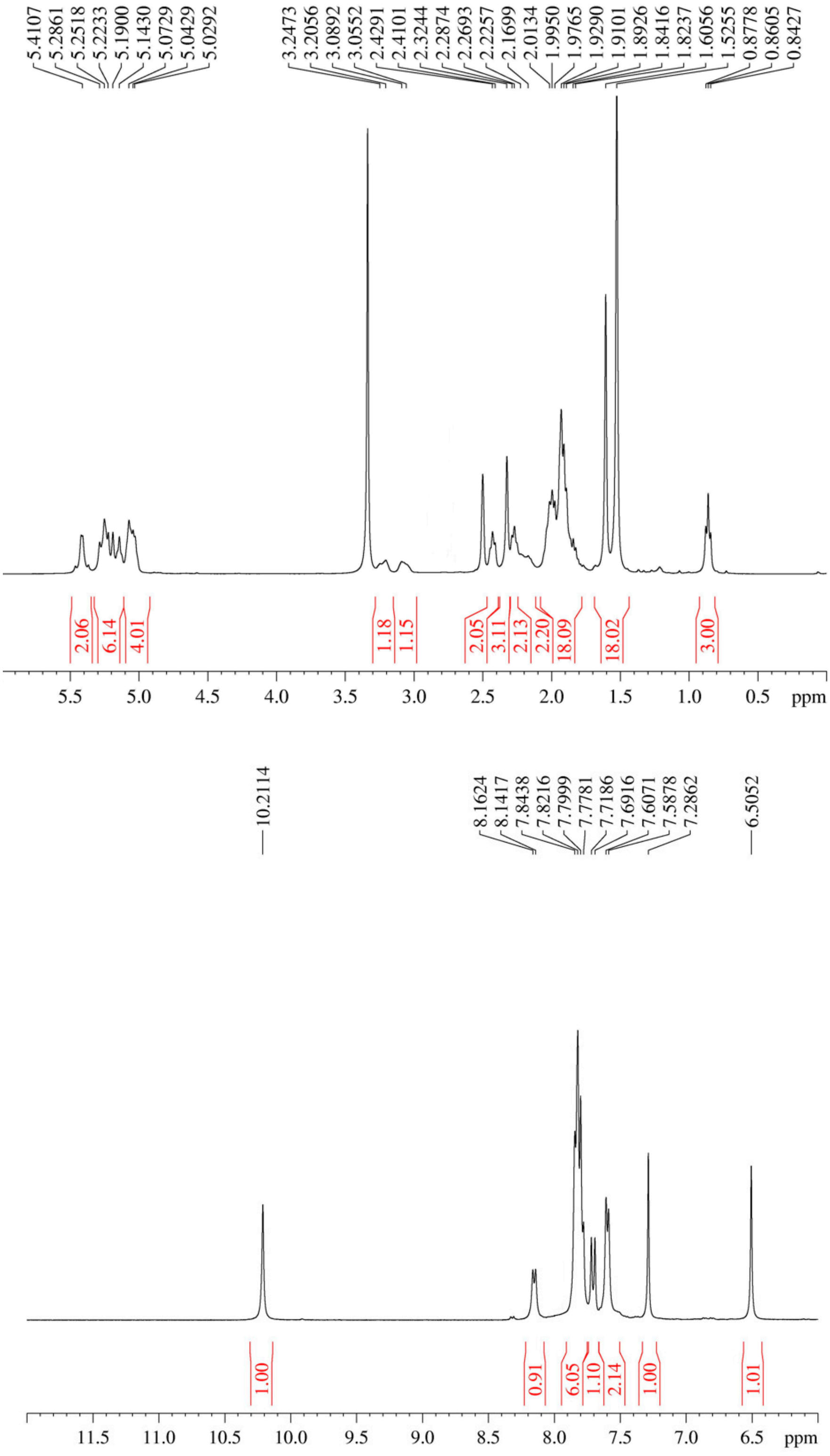

Figure S3. The ^{1}H NMR spectrum of EXA-PRO.

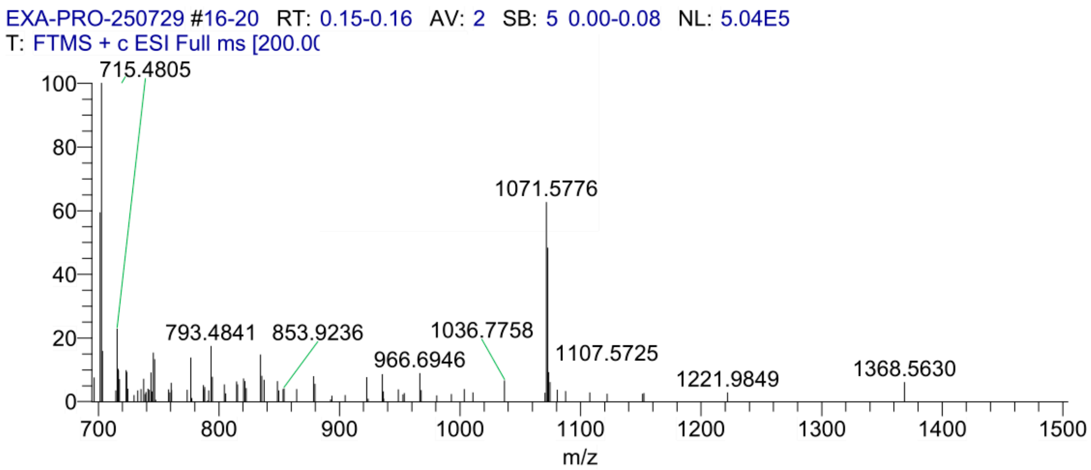

Mass	Calc. Mass	mDa	PPM	RDB equiv	Formula
1071.5776	1071.5754	2.2	2.013	30.5	C65H76FN6O7

Figure S4. The MS spectrum of EXA-PRO.

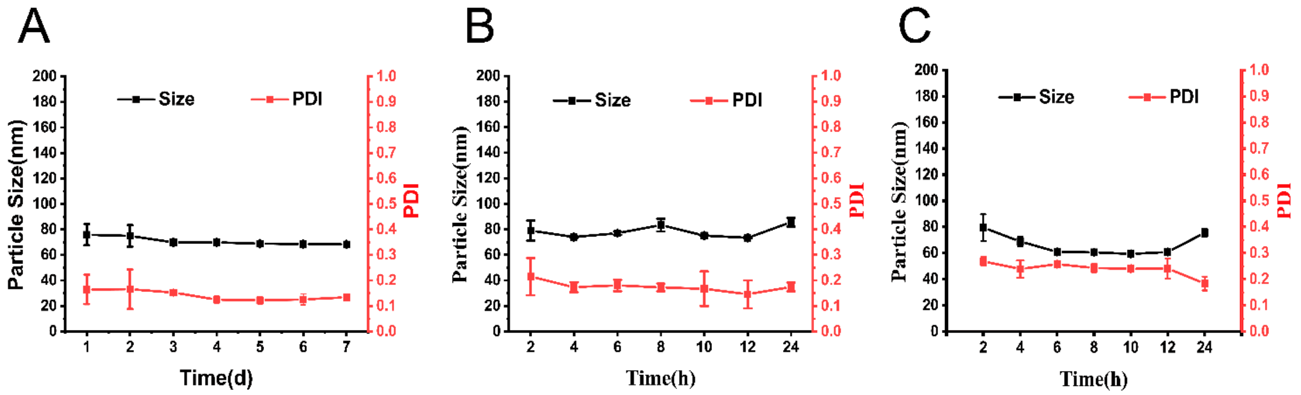

Figure S5. Stability of DP-EP NPs in Different Media. **(A)** Changes in particle size and PDI over 7 days at 4°C; **(B)** Changes in particle size and PDI within 24 h in PBS containing 10% FBS at 37°C; **(C)** Changes in particle size and PDI within 24 h in DMEM at 37°C.

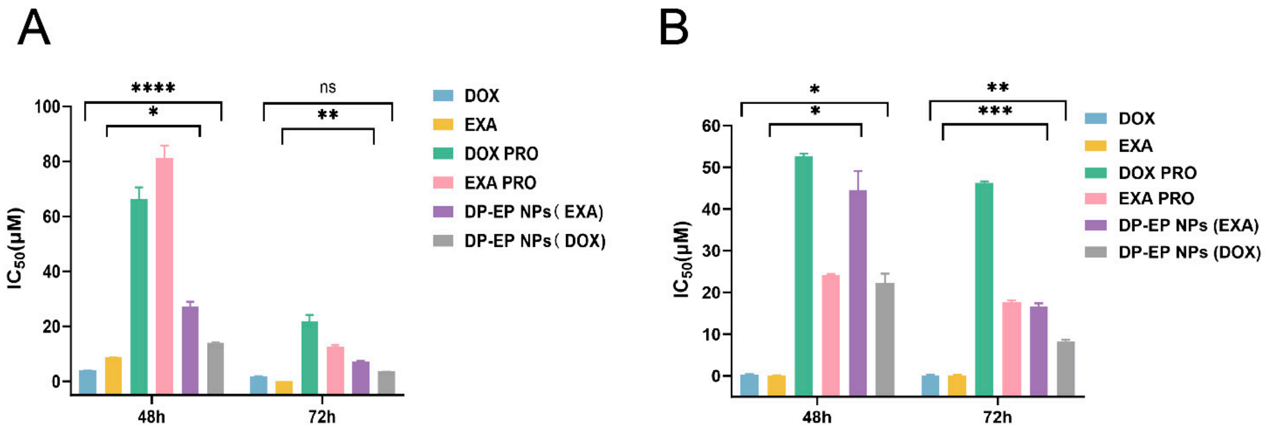

Figure S6. IC_{50} Values of Different Administration Groups on 4T1 **(A)** and MCF-7 **(B)** Cells at Different Time Points.

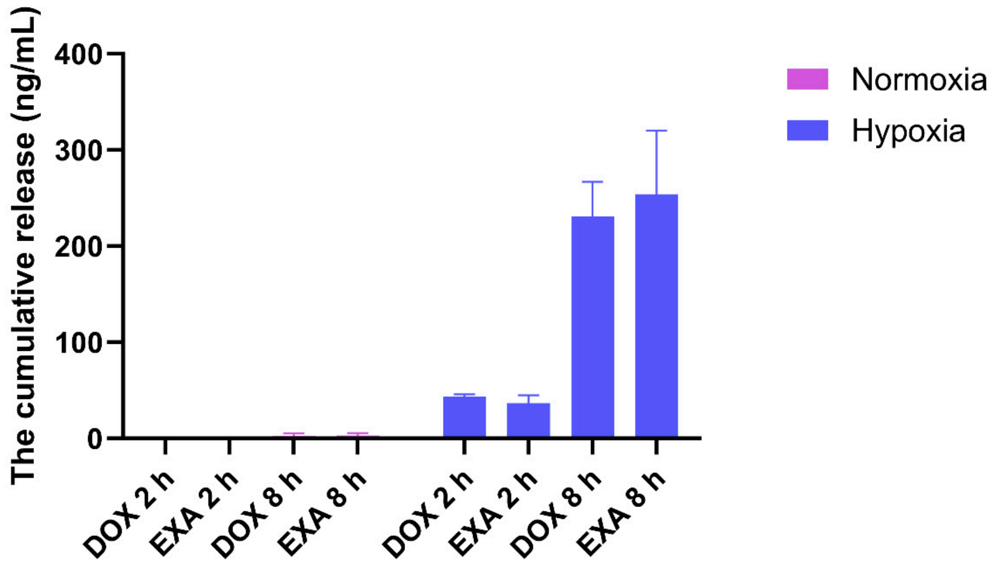

Figure S7. Intracellular release of DOX and EXA from prodrug nanoparticles under normoxia and hypoxia.

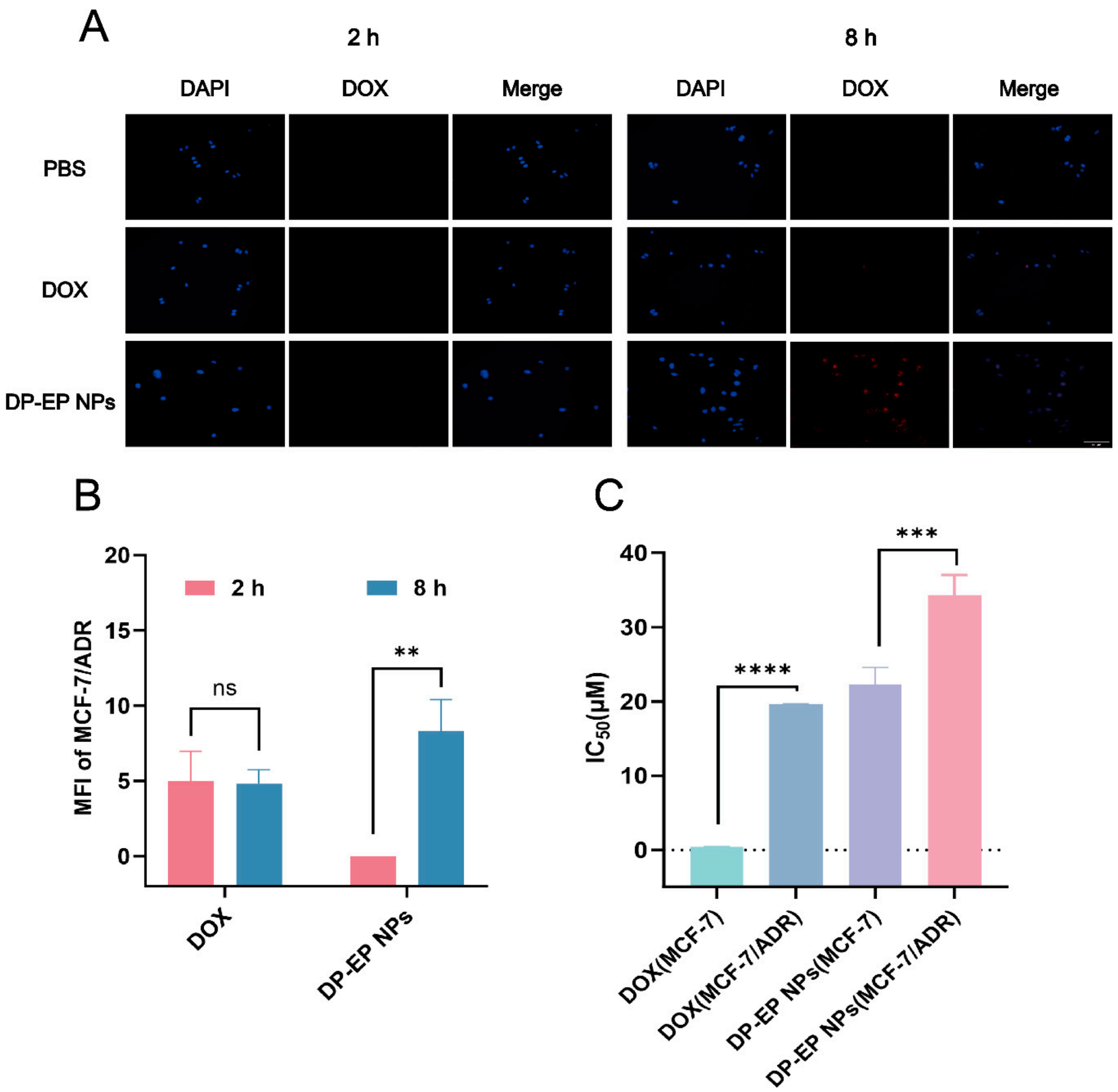

Figure S8. **(A)** Doxorubicin (DOX) Uptake Under Hypoxic Conditions (MCF-7/ADR Cells); **(B)** Fluorescence Quantitative Results by Image J; **(C)** MCF-7/ADR Cell Viability After Administration in Different Treatment Groups (72 h).

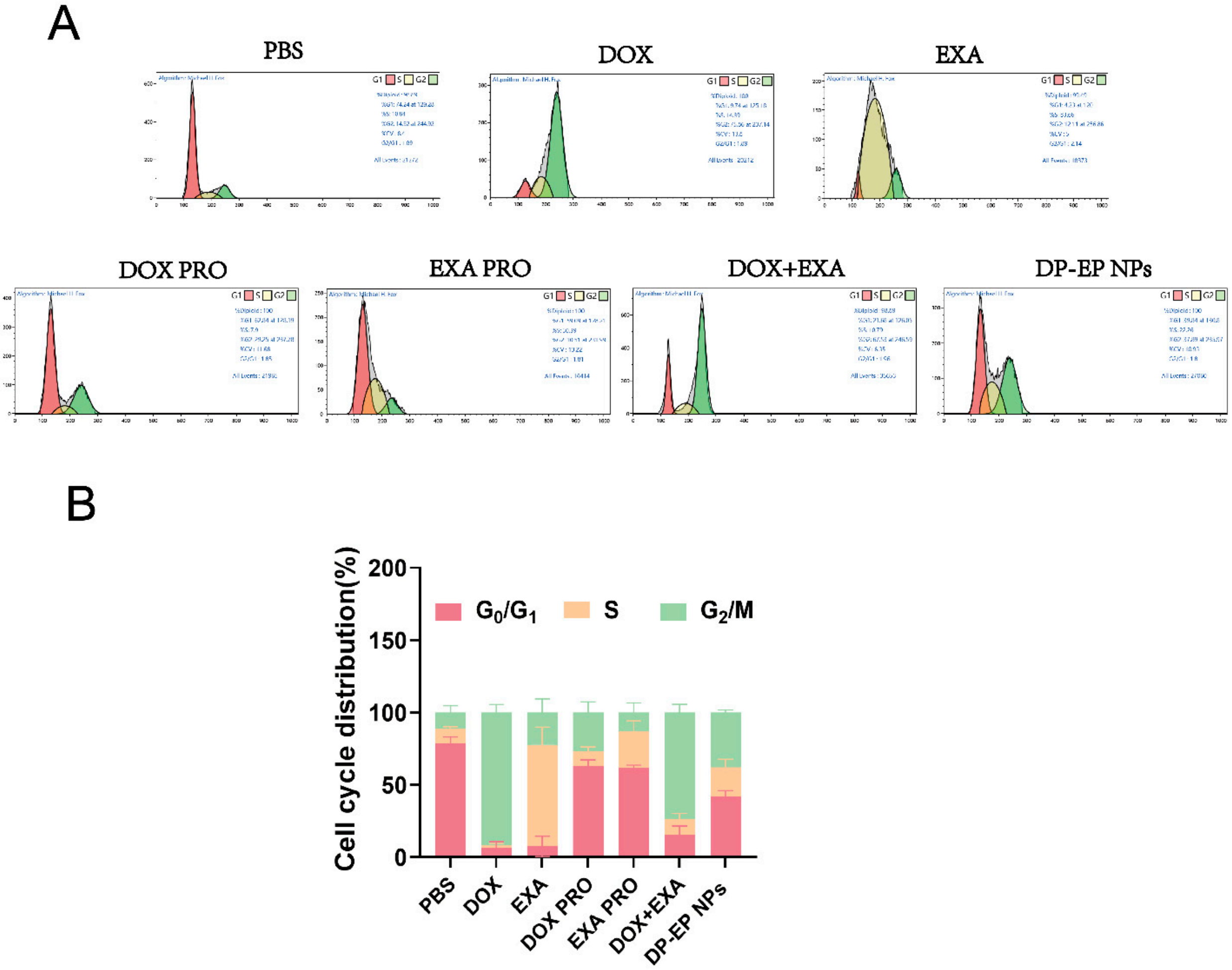

Figure S9. Different Administration Groups of 4T1 Cells. **(A)** Cell Cycle Arrest Profiles; **(B)** Stacked Histogram of Cell Cycle Distribution Percentages.

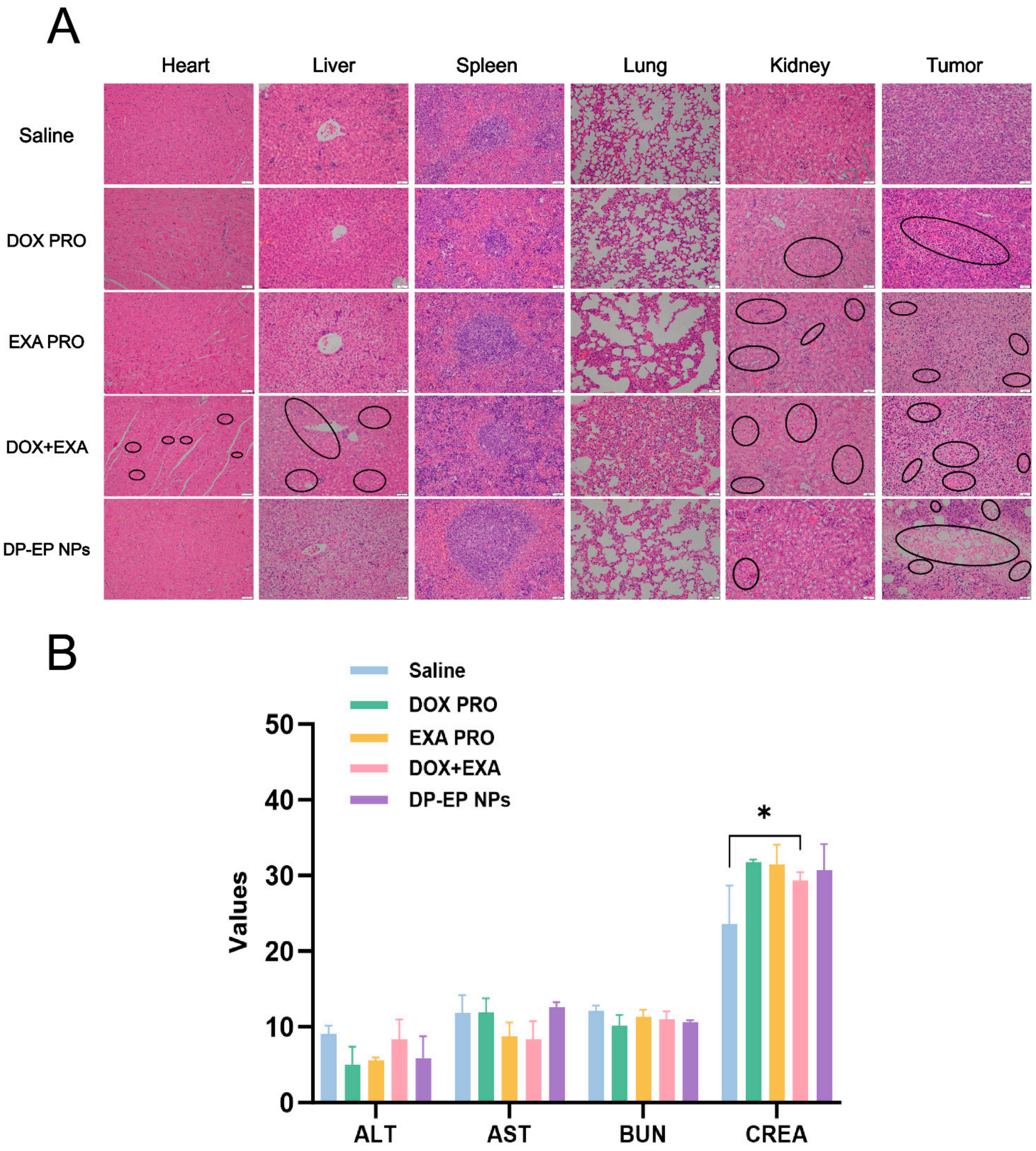

Figure S10. Tolerability evaluation. (A) H&E-stained sections of major organs and tumors (scale bar, 50 μm); and (B) serum biochemical indices of liver and kidney function.
